# Early Post-Vaccination Gene Signatures Correlate With the Magnitude and Function of Vaccine-Induced HIV Envelope-Specific Plasma Antibodies in Infant Rhesus Macaques

**DOI:** 10.3389/fimmu.2022.840976

**Published:** 2022-04-27

**Authors:** K. K. Vidya Vijayan, Kaitlyn A. Cross, Alan D. Curtis, Koen K. A. Van Rompay, Justin Pollara, Christopher B. Fox, Mark Tomai, Tomáš Hanke, Genevieve Fouda, Michael G. Hudgens, Sallie R. Permar, Kristina De Paris

**Affiliations:** ^1^ Department of Microbiology and Immunology, Center for AIDS Research, and Children’s Research Institute, School of Medicine, University of North Carolina at Chapel Hill, Chapel Hill, NC, United States; ^2^ Department of Biostatistics, Gillings School of Public Health, University of North Carolina at Chapel Hill, Chapel Hill, NC, United States; ^3^ California National Primate Research Center, University of California, Davis, Davis, CA, United States; ^4^ Duke Human Vaccine Institute, Duke University Medical Center, Durham, NC, United States; ^5^ Departent of Surgery, Duke University School of Medicine, Durham, NC, United States; ^6^ Department of Molecular Genetics and Microbiology, Duke University Medical Center, Durham, NC, United States; ^7^ Infectious Disease Research Institute, Seattle, WA, United States; ^8^ 3M Corporate Research Materials Laboratory, Saint Paul, MN, United States; ^9^ The Jenner Institute, University of Oxford, Oxford, United Kingdom; ^10^ Joint Research Center for Human Retrovirus Infection, Kumamoto University, Kumamoto, Japan; ^11^ Department of Pediatrics, Weill Cornell Medical College, New York, NY, United States

**Keywords:** systems biology, innate gene signatures, vaccine-induced antibody response, early life HIV vaccine, rhesus macaque model

## Abstract

A better understanding of the impact of early innate immune responses after vaccine priming on vaccine-elicited adaptive immune responses could inform rational design for effective HIV vaccines. The current study compared the whole blood molecular immune signatures of a 3M-052-SE adjuvanted HIV Env protein vaccine to a regimen combining the adjuvanted Env protein with simultaneous administration of a modified Vaccinia Ankara vector expressing HIV Env in infant rhesus macaques at days 0, 1, and 3 post vaccine prime. Both vaccines induced a rapid innate response, evident by elevated inflammatory plasma cytokines and altered gene expression. We identified 25 differentially-expressed genes (DEG) on day 1 compared to day 0 in the HIV protein vaccine group. In contrast, in the group that received both the Env protein and the MVA-Env vaccine only two DEG were identified, implying that the MVA-Env modified the innate response to the adjuvanted protein vaccine. By day 3, only three DEG maintained altered expression, indicative of the transient nature of the innate response. The DEG represented immune pathways associated with complement activation, type I interferon and interleukin signaling, pathogen sensing, and induction of adaptive immunity. DEG expression on day 1 was correlated to Env-specific antibody responses, in particular antibody-dependent cytotoxicity responses at week 34, and Env-specific follicular T helper cells. Results from network analysis supported the interaction of DEG and their proteins in B cell activation. These results emphasize that vaccine-induced HIV-specific antibody responses can be optimized through the modulation of the innate response to the vaccine prime.

## Introduction

Novel antiretroviral treatment (ART) options and improved prevention services have resulted in a major decline of new HIV infections in the last decade. Yet, the 90-90-90 goals have not been reached, with 10 million people living with HIV (>25%) still not receiving ART (1). The number of new HIV infections, 1.5 million globally, was three times as high as prioritized in the United Nations Sustainable Development Goals for 2020. In Eastern Europe and central Asia, new HIV infections have increased by >70% since 2010 (1). In sub-Saharan Africa, young women aged 15-24 years accounted for 25% of new HIV infections in 2020 although they only represent 10% of the population (1). Two fifths of all HIV-infected children (0-14 years) remain undiagnosed and only 40% of children with known HIV status and receiving ART are fully suppressed (1). These numbers emphasize the continuous and pressing need for an effective HIV vaccine to curb the pandemic, especially among young people. Our group is pursuing the idea that an HIV vaccine regimen started in early life - with booster immunizations in childhood - would provide the necessary time to mature vaccine-induced HIV-specific antibody responses that could protect against HIV acquisition in the high-risk group of adolescents prior to sexual debut.

Challenges in HIV vaccine development include the immense diversity of the virus, the difficulty in designing Env immunogens that can capture this diversity and present epitopes of vulnerability to the immune system, and the possible need for strategies that can target the various arms of the immune system to induce protective immunity. Systems vaccinology approaches, including transcriptomics, plasma proteomics, structure-based immunogens and rational adjuvant design, have emerged as important tools to inform vaccine design and to predict vaccine immunogenicity and efficacy (2–14). Notably, retrospective analyses of vaccine trials have demonstrated that innate immune responses induced by the vaccine prime impact the subsequent vaccine-induced adaptive immunity (6, 8–14). As the infant immune system is highly dynamic in the first few months of life, it is important to determine if early immune signatures induced by the vaccine prime can also predict immunogenicity and/or efficacy in pediatric vaccines. The goal of our current study was to determine whether early innate immune responses after the vaccine prime were associated with functional antibody responses in the memory phase after immunization of infant rhesus macaques with two different HIV envelope (Env) vaccine regimens.

Our group has previously demonstrated that infant rhesus macaques can mount potent and persistent HIV Env-specific antibody responses to an HIV Env protein vaccine mixed with the TLR7/8-based 3M-052 adjuvant in stable emulsion (SE) and to a vaccine regimen consisting of both the adjuvanted Env protein and a modified Vaccinia Ankara vector expressing HIV Env (MVA-Env) (15, 16). In the current study, we determined plasma cytokine levels and the whole blood transcriptome at days 1 and 3 after the vaccine prime in comparison to day 0 and tested for correlations between early innate immune responses and later adaptive vaccine-induced cellular and humoral responses during the memory phase and in response to a late boost. Our results demonstrate a rapid, systemic innate response to the vaccine prime at day 1. The response was more pronounced in animals receiving the 3M-052-SE adjuvanted Env protein vaccine compared to the animals immunized simultaneously with the adjuvanted protein plus the MVA-Env vaccine. Several of the differentially expressed genes (DEG) on day 1 were positively correlated with Env-specific plasma IgG responses at week 14, and with Env-specific antibody-mediated cellular cytotoxicity (ADCC) and Env-specific follicular T helper cells (T_FH_) at week 34. In contrast, early molecular signatures were negatively correlated with HIV Env-specific CD8^+^ T cell responses. These findings imply that vaccine-induced HIV-specific immune responses could be optimized through targeted modulation of innate responses to the vaccine prime.

## Methods

### Study Design

**Figure 1:**
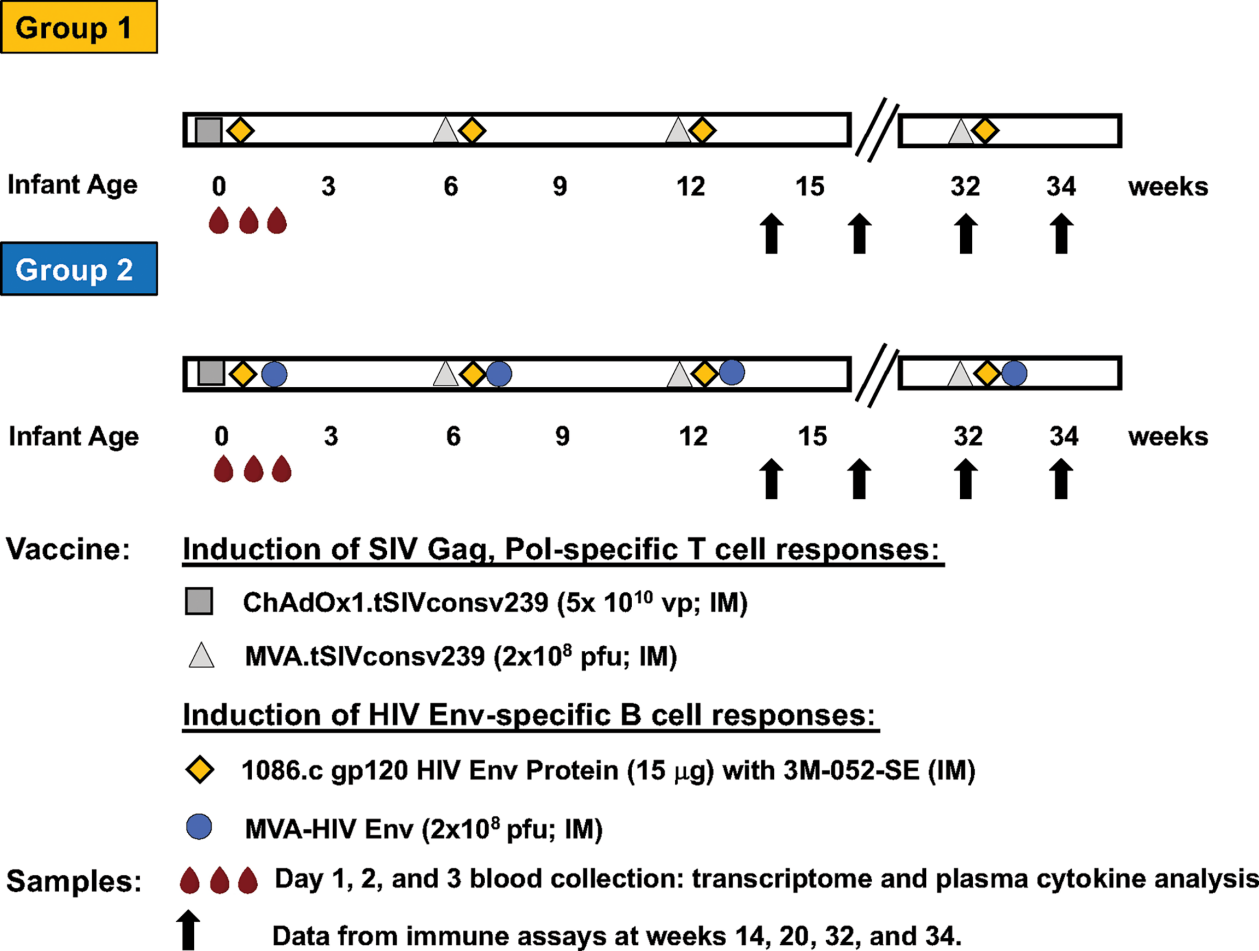
Vaccine study overview. The timeline of immunization with the specific vaccine regimens and timepoints of immunizations for Group 1 and Group 2 are illustrated in horizontal bars. Each vaccine is indicated by a distinct symbol; vaccine dose and route of administration are listed in the legend. Blood samples (red droplets) are the vaccine prime were collected at days 0 (immediately prior to immunization) and at days 1 and 3 post prime. Vaccine-induced adaptive immune responses were measured at weeks 14, 20, 32 and 34, with results having been reported previously (15, 16).

The current study utilized whole blood samples from a previously reported vaccine study in infant rhesus macaques (15, 16). Study design, sample collection, and sample processing have been described in detail previously (15, 16). Briefly, infant RMs were immunized during the first week of life with (i) 15 μg 1086.c HIV Env protein administered intramuscularly (IM) with 10 μg 3M-052 adjuvant in 2% v/v stable emulsion (Group 1 or Protein group; n=10), or with (ii) Env Protein in 3M-052-SE and 10^8^ pfu MVA expressing 1086.c Env (Group 2 or MVA/Protein group; n=10). In addition, both groups received an IM immunization with the Chimpanzee Adenovirus vector Ox1t that expresses conserved regions of SIV Gag/Pol to promote SIV-specific T cell responses on day zero (D0) (17). ChAdOx1.tSIVconsv239 (5x10^10^ virus particles [vp]) immunizations were divided equally over the left and right gluteus (15, 16) (**Figure 1**). Animals in both groups received booster immunizations at weeks 6 and 12 and a late boost at week 32 (15, 16). The vaccine boosts were identical to the vaccine prime, except for the use of MVA.tSIVconsv239 (10^8^ pfu) as boost for the initial ChAdOx1.tSIVconsv239 prime (15, 16) (**Figure 1**). As reported previously, animals were euthanized at week 34 to analyze vaccine-induced immune responses in blood, lymph nodes, and in intestinal tissues (15, 16).

To summarize, Group 1 and Group 2 animals received the same vaccine to induce SIV Gag/Pol-specific T cell responses but differed in the vaccine components aimed at inducing HIV Env-specific antibody responses (**Figure 1**).

### Whole Blood RNA Isolation and Gene Expression Analysis With NanoString nCounter^®^

For the purpose of the current study, EDTA-anticoagulated venous blood samples (3 x 200 μl) were collected on day 0 (= vaccine prime) just prior to immunization and at days 1 (24 hrs) and 3 (72 hrs) post prime and immediately resuspended in PAXgene reagent (552 μl). Samples were incubated for 2 hrs at room temperature and then stored at -80^0^C until analysis.

RNA was extracted using the PAXgene Blood RNA kit (PreAnalytix GmbH, Hombrechtikon, Switzerland) following the manufacturer’s protocol, except for incubating the samples for 60 minutes at 55**^0^** C - instead of the recommended 10 minutes - after the addition of BR2 and Proteinase K. The extracted RNA was further purified with the RNA Clean and Concentrator Kit (Zymo Research Cooperation, Irvine, CA, USA). RNA was quantified using the Qubit RNA HS assay (ThermoFisher, Waltham, MA, USA). We obtained sufficient RNA for n=17 day 0, n=17 day 1, and n=14 day 3 samples to proceed with transcriptome analysis (**Table 1**). RNA samples (50 ng) were analyzed with the Nanostring Non-Human Primate Immunology Panel comprised of 754 immune-related genes and 16 internal reference genes. Gene expression analysis was conducted according to the manufacturer’s protocol utilizing the NanoString nCounter^®^ Prep Station and NanoString nCounter^®^ Digital Analyzer.

**Table 1:** List of animals and samples for transcriptome analysis.

Group	Animal No.	Sample Availability		
		Day 0	Day 1	Day 2
1	RM1	x	x	x
	RM2	x		
	RM3	x	x	x
	RM4	x	x	x
	RM5	x	x	
	RM6	x	x	x
	RM7			x
	RM8		x	x
	RM9	x	x	
	Total:	n=7	n=7	n=6
2	RM10	x	x	x
	RM11	x	x	x
	RM12		x	x
	RM13	x	x	x
	RM14	x		x
	RM15	x	x	
	RM16	x	x	x
	RM17	x	x	x
	RM18	x	x	x
	Total:	n=8	n=8	n=8

### Gene Expression Data Analysis

Raw gene expression data across days 0, 1, and 3 were analyzed using the NanoString^®^ software nSolver v3.0.22 with the Advanced Analysis Module v2.0. The raw data files underwent quality evaluation applying nSolver Imaging and Binding Density Quality Control (QC) metrics, checking for Positive Control Linearity, and assessing Limit of Detection parameters. One day 0 (D0) sample (Group 1 RM 8) was flagged for low binding density and low limit of detection and therefore removed from further analysis. To delineate false positives, background correction was performed using a threshold value of 20; samples with counts <20 being adjusted to the value 20. Genes with altered expression levels on D1 or D3 compared to D0 were identified utilizing the Advanced Analysis Module v2.0 in nSolver™ that uses open-source R programs for QC, normalization, Differential Expression (DE) analysis, pathway scoring, and gene-set enrichment analysis. Data normalization employs the geNorm algorithm (18) through the function selectHKs in the Bioconductor package NormqPCR. The overall sample quality was represented by an assigned normalization factor and mean squared error (MSE). One D0 sample (Group 2 RM 12) and two day 1 (D1) samples (RM2 and RM7, both Group 1) had high MSE values far distinct from other samples and were, thus, designated as outliers and excluded from further analysis. Therefore, we had a total of n=15 D0, n=15 D1, and n=14 D3 samples for analysis (**Table 1**).

Genes with altered expression levels on D1 or D3 compared to D0 were identified employing multivariate linear regression models; raw p values were adjusted by Benjamini-Yekutieli method to minimize the false discovery rate. DEG were defined as having a log_2_ fold-change ≥1.32 (or 2.5-fold linear change) in expression and an adjusted p-value ≤0.1. ClustVis (http://biit.cs.ut.ee/clustvis/) was used for principal component analysis (PCA) using log_2_ transcript count values. In addition, we utilized nSolver™ to generate pathway scores to define potential immune pathways altered by the innate response to the vaccine prime. Pathway scores are based on the first principal component of the normalized relative gene expression of genes belonging to a specific immune pathway. The scores are further standardized by Z scaling. Therefore, pathway scores can have positive or negative values.

Gene expression data have been uploaded to Gene Expression Omnibus (GEO) at NCBI (submission number GSE192584).

### Network Analysis

Network analyses for interactions of proteins encoded by differentially expressed genes on D1 compared to D0 were performed using the Search Tool for the Retrieval of Interacting Genes/Proteins (STRING) database version 11.5 (http://stringdb.org/), which curates both experimental and predicted protein interactions. Interactions with an interaction score >0.4 were visualized with Cytoscape v3.8.2 (www.Cytoscape.org/), with nodes representing significant genes/proteins and edge width indicating the combined interaction score. Protein-protein interactions were also visualized using NetworkAnalyst 3.0 (networkanalyst.ca) (19), an open source software, that utilizes the Human Interactome of the STRING v11.5 database (20).

### Multiplex Cytokine Analysis

Plasma cytokine concentrations were measured using a custom-designed NHP Procartaplex Mix and Match 14-plex (ThermoFischer Scientific Inc) consisting of granulocyte-monocyte-colony stimulating factor (GM-CSF), interferon alpha (IFN-α), IFN gamma (IFN-γ), interleukin 1 beta (IL-1β), IL-1 receptor antagonist (IL-1RA), IL-6, IL-8 (CXCL8), IL-10, IL-12p70, IL-18, IL-23, interferon-inducible protein 10 (IP-10 or CXCL10), monocyte-chemoattractant protein 1 (MCP-1 or CCL2), and tumor necrosis factor alpha (TNF-α). Data were acquired on MAGPIX instrument with Luminex xPONENT software version 4.2. Cytokine concentrations were determined using ProcartaPlex Analyst software version1.0.

### Statistical Analysis

Plasma cytokine concentrations of Group 1 or Group 2 animals on D1 or D3 were compared to D0 plasma cytokine concentrations by Mann-Whitney test using GraphPad Prism version 9.0, with p<0.05 being considered significant. Similarly, differences in mRNA expression or in pathway scores of Groups 1 or Group 2 animals on D1 compared to D0 were assessed by Mann-Whitney test.

To test for correlations between early gene signatures and/or plasma cytokines and adaptive immune responses at later timepoints, we combined data from Group 1 and Group 2 animals for D1 and D3. This sample size provides 80% power to detect a Spearman correlation of 0.7, and 66% power to detect a Spearman correlation of 0.5 at the α = 0.05 level. To adjust for multiple comparisons, the Benjamini–Hochberg (BH) procedure was used to control the false discovery rate (FDR). Adjustments to control the FDR at α = 0.05 were performed separately for humoral and cellular immune responses. Humoral responses included Env-specific plasma IgG responses (weeks 14, 20, 32 and 34), ADCC responses (weeks 14, 20, 32, and 34), and neutralizing antibody titers (weeks 14, 32, and 34) for a total of 661 correlation tests. Cellular responses included total peripheral blood and lymph node memory B cells and lymph node germinal center B cells (week 34), Env-specific follicular T helper cell (T_FH_) responses (week 34), and HIV Env and SIV Gag specific CD8^+^ T cell responses (week 34) for a total of 366 correlation tests. Spearman rank correlation coefficients between early mRNA expression and/or plasma cytokines and vaccine-elicited adaptive immune parameters were calculated, tested, and FDR adjustments were performed using SAS version 9.4 (Cary, NC, USA).

## Results

### Overview of the Study Design

The current study leveraged samples and vaccine-induced immune response data from a previously reported pediatric HIV vaccine study in infant rhesus macaques that was comprised of 2 groups, each with 10 animals (15, 16). On day 0 (D0), animals in both groups received an IM immunization with ChAdOx1.tSIVconsv239 expressing conserved Gag/Pol epitopes (17) to promote SIV Gag/Pol-specific T cell responses (**Figure 1**). Both vaccine groups received booster immunizations with MVA.tSIVconsv239 (10^8^ pfu) at weeks 6 and 12, and a late boost at week 32 (15, 16) (**Figure 1**). The two vaccine groups differed in the component designed to induce HIV envelope-specific antibody responses. Animals in Group 1 were vaccinated with 1086.c Env protein administered IM with 3M-052 adjuvant in stable emulsion and Group 2 was immunized with the same adjuvanted HIV Env protein vaccine and with modified Vaccinia Ankara expressing 1086.c Env (MVA-Env) (15, 16). HIV Env protein was given IM into the left and right quadriceps and MVA-Env was administered IM into the left and right biceps (**Figure 1**). Blood was collected just prior to immunization (D0 or baseline), and on days 1 (D1) and 3 (D3) after the initial immunization (vaccine prime) (**Figure 1**). Animals in both vaccine groups received booster immunizations identical to the vaccine prime at weeks 6 and 12, and a late boost at week 32 (15, 16) (**Figure 1**).

Changes in soluble immune mediators in plasma in response to the vaccine prime were measured by multiparameter bead arrays and changes in gene expression were determined using the Nanostring^®^ NHP Immunology Panel. Innate immune responses were correlated to previously reported vaccine-induced HIV 1086.c Env-specific IgG responses at the peak of the antibody responses after the initial 3 immunizations (week 14), during the memory phase of vaccine-induced antibody responses at weeks 20 and 32, and two weeks after the late boost (week 34) (15, 16). We also tested for correlations between innate responses induced by the vaccine prime and cellular immune responses at week 34, including total memory B cells, germinal center (GC) B cells in lymph nodes, Env-specific follicular T helper cells (T_FH_), and HIV Env- or SIV Gag-specific CD8^+^ T cell responses (**Figure 1**; see **Supplemental Figures S1**-**3** for flow cytometry gating strategies).

### Impact of Vaccination on Soluble Immune Mediators in Plasma

**Figure 2:**
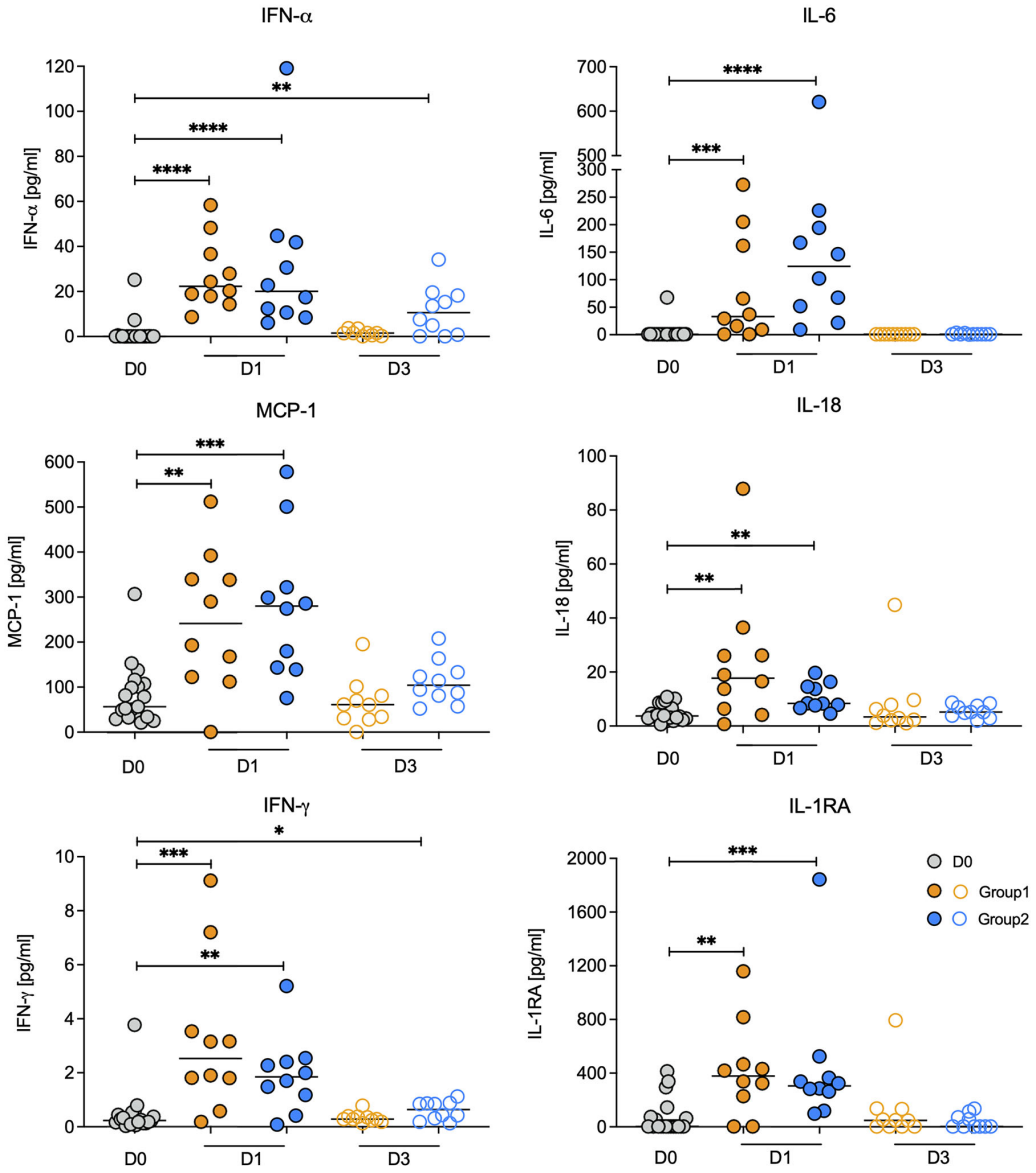
Changes in plasma cytokine concentrations post vaccination. Plasma cytokine concentrations for IFN-a, IL-6, IL-18, IFN-g, MCP-1, and IL-1RA on day 0 (vaccine prime; grey circles) and on days 1 (filled circles) and day 3 (open circles) post vaccination for Group 1 (orange circles) and Group 2 (blue circles) animals. Note that D0 includes animals from both Group 1 and Group 2. Horizontal lines represent median values. Data between two time points were compared by Mann-Whitney test with *, **, ***, **** indicating p<0.05, p<0.01, p<0.001, or p<0.0001 respectively.

To assess the systemic effect of the vaccine prime, we measured 14 immune mediators on days 0, 1, and 3 in plasma. On D1, the proinflammatory cytokines IFN-α, IL-6, IL-18, IFN-γ, MCP-1 (aka CCL2) and the anti-inflammatory cytokine IL-1RA were increased in both groups and at a similar magnitude (**Figure 2**). By D3, all 6 cytokines had returned to baseline levels in Group 1, whereas in Group 2 IFN-α and IFN-γ remained slightly elevated compared to D0 (**Figure 2**). These results implied that both vaccine regimens induced a transient inflammatory response.

**Figure 3:**
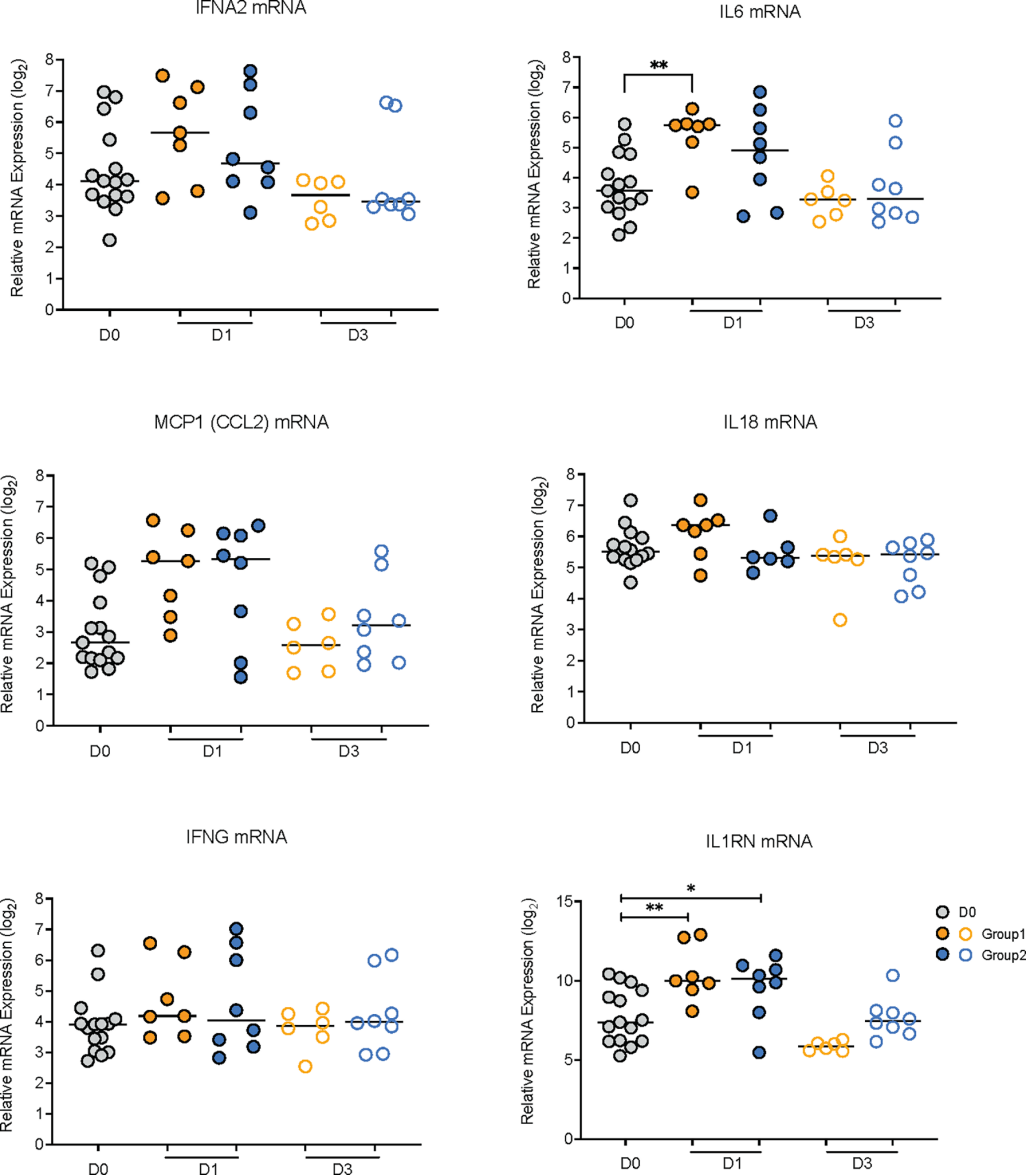
Relative mRNA expression values of genes encoding elevated plasma cytokines. The graphs show relative log_2_ mRNA levels of IFNA2, IL6, MCP1, IL18, IFNG, and IL1RN on day 0 (vaccine prime; grey circles) and on days 1 (filled circles) and day 3 (open circles) post vaccination for Group 1 (orange circles) and Group 2 (blue circles) animals. Horizontal lines represent median values. Data between two time points were compared by Mann-Whitney test with * or ** indicating p < 0.05 or p < 0.01, respectively.

To further interrogate this point, we determined whether the mRNA levels of the corresponding genes of the elevated plasma cytokines were also increased on D1 compared to D0. The mRNA levels of IL1RN, the gene encoding IL-1RA, were increased on D1 in both groups (**Figure 3**). In Group 1, IL6 mRNA levels were also increased on D1 and there was a trend towards higher median mRNA levels of IFNA2, IL18, and MCP1, but these did not reach statistical significance (**Figure 3**). On D3, consistent with a transient inflammatory response, mRNA levels of all six cytokines were indistinguishable from D0 mRNA levels in animals of both groups (**Figure 3**).

### Differential Gene Expression in Response to the Vaccine Prime

We performed whole blood transcriptomics analysis to define the genes and immune pathways associated with immune activation by the two vaccine regimens. We first compared D0 mRNA expression levels of animals in Group 1 and Group 2 to confirm that baseline parameters did not differ between the two groups. Applying principal component analysis (PCA), our results demonstrated that the D0 transcript profile of the animals from Group 1 (n=8) was indeed similar to the transcript profile of Group 2 animals (n=7) (**Supplemental Figure S4**). Therefore, we combined the D0 mRNA expression data from Group 1 and Group 2 (n=15) in subsequent analyses to assess changes in gene expressions on D1 and D3 in response to the vaccine prime on D0, and to determine whether the early post-prime innate gene signatures of Groups 1 and 2 differed dependent on the vaccine regimen.

**Figure 4:**
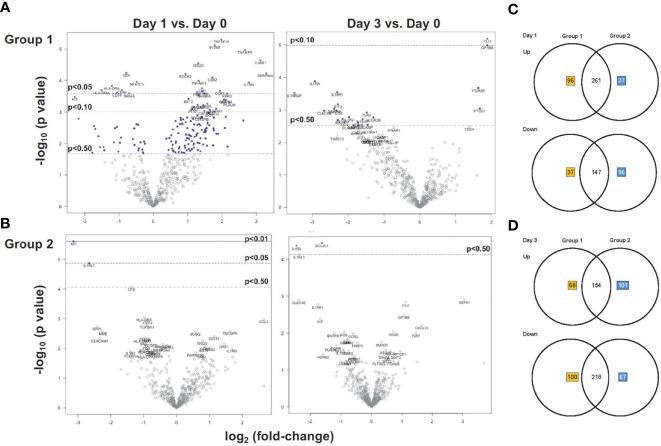
Gene expression analysis of Group 1 and Group 2 prior and post vaccination. **(A, B)** Volcano plots displaying the log_2_ change in mRNA expression on D1 (left plots) and D3 (right plots) versus D0 in Group 1 **(A)** and Group 2 **(B)**. The x-axis lists the log_2_ fold-change and the y-axis the corresponding -log_10_ p-value for each gene (each gene is represented by a circle). Dashed horizontal lines indicate the adjusted p-value thresholds of p < 0.05 and p < 0.1 determined by Benjamini-Yekutieli procedure. Genes highlighted by red boxes indicate representative examples of the identical gene in Group 1 and Group 2 with the same direction (up- or down-regulation) in the change of mRNA expression on day1 (left plots) or day 3 (right plots). **(C, D)** Venn diagrams depicting the number of unique and shared up-regulated and downregulated genes at D1 **(A)** and D3 **(B)** after vaccination in Groups 1 and 2. Unique genes in Group 1 or Group 2 are indicated by orange or blue numbers respectively, the number of shared genes is listed in black.

In Group 1, more than twice as many genes were upregulated (n=375) than downregulated (n=164) on D1 compared to D0 (**Figure 4A**); the change in mRNA levels of several of these genes (n>20) reached an adjusted p value of p<0.05 (**Figure 4A**). By D3, an opposite trend was noted, with most genes (n=318) being downregulated in Group 1. In Group 2, about an equal number of genes were up- or down-regulated on D1 and D3 compared to D0 (**Figure 4B**). In contrast to Group 1, no genes were induced with adjusted p<0.05 on D1 or D3 in Group 2. This result implied that the MVA-Env vaccine in Group 2 may have altered the innate response induced by the 3M-052-SE adjuvanted Env Protein vaccine that was administered to both groups. Nonetheless, there was large overlap between Group 1 and Group 2 in the number of genes that were up- or down-regulated on D1 and D3 (**Figures 4C, D**).

**Figure 5:**
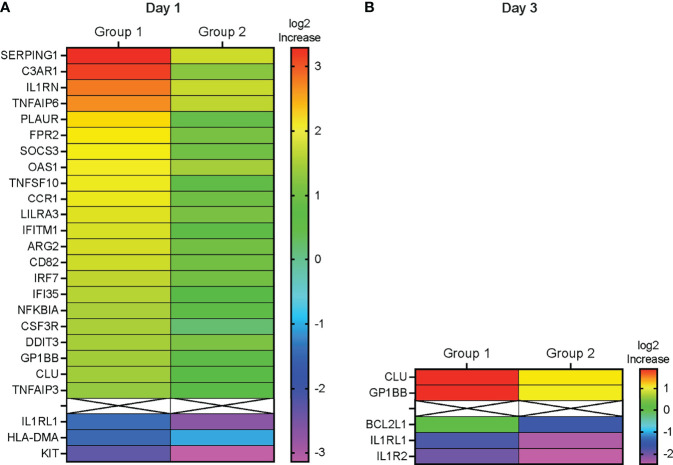
Differentially expressed genes. **(A, B)** Heatmap depicting the log_2_ fold-increase (FI) of differentially expressed genes at D1 compared to D0 **(A)** or at D3 compared to D0 **(B)** with an adjusted p≤ 0.1. DEG are ordered from top to bottom according the highest (red) to the lowest (purple) fold-increase (FI) in log2 gene expression in Group 1 as indicated by the color legend bar. The corresponding Group 2 log_2_ gene expression values are also shown.

To identify the genes that had undergone the greatest increase or decrease in mRNA expression in response to the vaccine prime, we applied the combined criteria of a ≥1.32 log_2_ (or 2.5-fold change) increase or decrease in mRNA levels on D1 or D3 compared to D0 and the gene expression change having an adjusted p ≤ 0.1. On D1, we identified 22 DEG in Group 1 that were upregulated and 3 genes that were downregulated (**Figure 5**). In Group 2, only two genes fulfilled these criteria on D1, and both of these genes (KIT and IL1RL1) were downregulated (**Figure 5**). Increased mRNA expression appeared to be transient and only two of the DEG with higher mRNA expression levels on D1 (CLU and GP1BB) still had increased mRNA levels on D3 in Group 1 (**Figure 5**). Among the D1 downregulated DEG, IL1RL1 also had decreased mRNA levels on D3 (**Figure 5**). Two additional downregulated genes in Group 2, IL1R2 and BCL2L1, fulfilled DEG criteria (**Figure 5**).

Consistent with elevated plasma levels of IFN-α on D1 (**Figure 2**), interferon-inducible genes (e.g., OAS3, IRF7, IFITM1, IFI35, SOCS3, TNFIAP3, NFKBIA) represented a large number of D1 DEG. Furthermore, the DEG IL1RN encodes IL-1RA, one of the plasma cytokines that were elevated on D1 (see **Figure 2**). Other proteins encoded by DEG included complement activation factors (e.g., C3AR1), proteins associated with interleukin signaling (e.g., IL1RN, IL1RL1, NFKBIA), genes encoding inflammatory mediators (e.g., TNFSF10), chemotactic molecules (e.g., CCR1), and mediators of monocyte and dendritic cell activation (e.g., CSF3R) (**Supplemental Table S1**). KIT encodes a receptor tyrosine kinase III that is expressed on most hematopoietic cells and has been suggested to interfere with dendritic cell activation by T helper 1 cytokines (21, 22). Of the 25 D1 DEG, 19 could be integrated into a molecular interaction network (**Supplemental Figure S2**). For the remaining 6 genes (LILAR3, ARG2, CD82, DDIT3, GP1BB, and HLA-DMA) no direct interactions with the other DEG could be identified. Major hubs included IL1RN, NFKBIA, and IRF7 with 7 links each, followed by SOCS3 and TNFAIP3 (both 6 links), and by TNFSF10 and C3AR1 with 5 links (**Supplemental Figure S2**). The low number of DEG on D3 did not allow for the assembly of a molecular network and further emphasized that, although the vaccine prime induced an innate response, the response was transient in nature.

### Immune Pathway Analysis

**Figure 6:**
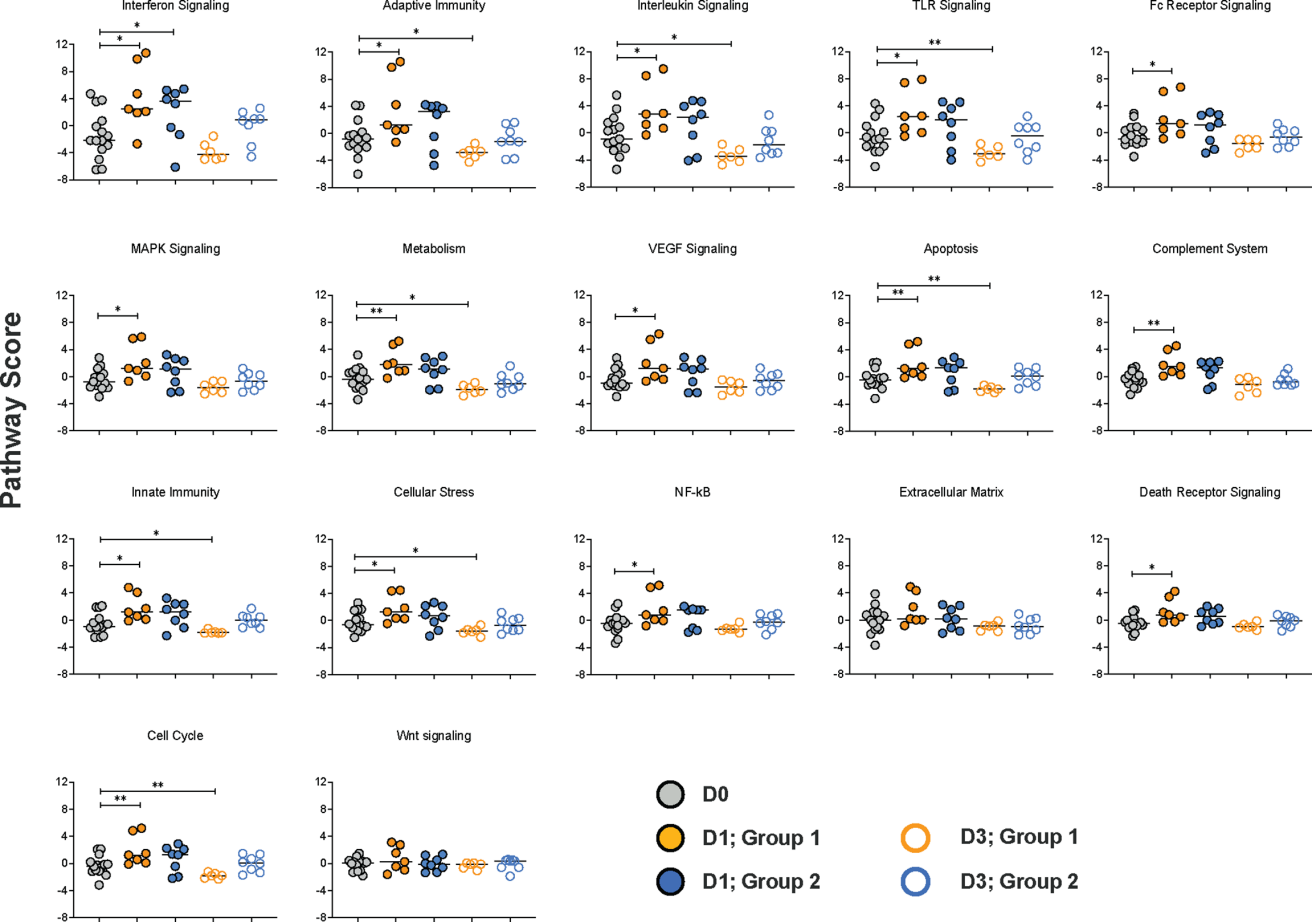
Immune pathways affected by the vaccine prime. Based on the first principal component of the normalized relative gene expression and the number of genes belonging to a specific immune pathway, a pathway score standardized by Z scaling was generated by the nSolver™ software. Each pathway is represented by a specific graph displaying the pathway scores on D0 (grey circles), and on days 1 (filled circles) and day 3 (open circles) post vaccination for Group 1 (orange circles) and Group 2 (blue circles) animals. Differences in pathway scores were determined by Mann-Whitney test with * and ** representing p < 0.05 or p < 0.01, respectively.

To gain more insights into the biological functions of the DEG, we performed pathway analysis utilizing nSolver™. Pathway scores are based on the first principal component of the normalized relative gene expression and the number of genes belonging to a specific immune pathway. Therefore, pathway scores can have positive or negative values. Overall, the Group 1 vaccine regimen resulted in increased scores for 15 of the 17 pathways included in the NanoString NHP Immunology Panel on D1 (**Figure 6**). In contrast, in Group 2, only the score for the interferon signaling pathway was increased on D1 and by D3 pathway scores were indistinguishable from D0 scores (**Figure 6**). However, pathway scores for 8 of the signaling pathways were reduced in Group 1 on D3 compared to D0 (**Figure 6**).

**Figure 7:**
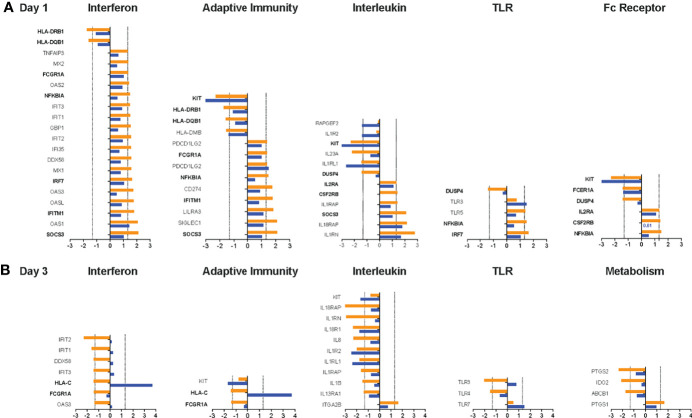
Changes in gene expression between Group 1 and Group 2 animals for the top pathways impacted by the vaccine prime. For each of the top 5 pathways, the mean log_2_ increase in mRNA levels on D1 compared to D0 **(A)** or D3 compared to D0 **(B)** is shown for genes within the pathway that had a log_2_ increase ≥1.3.2 or decrease (dashed lines) in Group 1 (orange bars). The mean change in mRNA levels of the same genes in animals of Group 2 is indicated by blue bars. Genes for each pathway are listed on the y-axis. Bolded genes indicate genes that are present in more than one pathway. 0.

To further interrogate how the two different vaccine regimens impacted these immune pathways, we compared the expression of genes within a specific pathway. Overall, the majority of genes within each of the pathways was detected in the transcriptome analysis (**Supplemental Table S3**). We focused on genes with a log_2_ increase or decrease ≥1.32 on D1 *vs* D0 (**Figure 7A**) or D3 *vs*. D0 (**Figure 7B**) in Group 1 to the mRNA expression of the same genes in Group 2 without considering whether the raw or adjusted p value was <0.05 (**Supplementary Table S4**). We selected the five pathways with the highest increase or decrease in pathway score on D1 or D3 compared to D0. Consistent with the finding that several of the DEG were part of the interferon signaling pathway, the highest D1 pathway score was observed for the Interferon Signaling Pathway (mean: 4.15). The adaptive immunity pathway was the next highest ranked pathway in (mean score: 3.65), followed by the interleukin (mean score: 3.60), Toll-like receptor (TLR) (mean score: 2.94), and the Fc receptor (FcR) (mean score: 2.25) signaling pathways. Upregulated genes on D1, with the exception of the TLR3 gene, were expressed at higher levels in Group1 compared to Group 2. The downregulated genes KIT and IL1RL1 were more strongly reduced in Group 2 compared to Group 1. Several genes with log_2_ ≥1.32 increased or decreased mRNA expression were represented in multiple pathways, indicative of some redundancy and crosstalk between the pathways. Some of the shared genes on D1 were part of the DEG (SOCS3, IFITM1, IRF7, NFKBIA, KIT), whereas other common genes (IL2RA, FCGRIA, HLA-DRB1, HLA-DQB1, CSF2RB, DUSP4) were not identified in the DEG analysis because the adjusted p value for the change in mRNA expression for D1 versus D0 was greater than 0.1.

An opposite picture emerged on D3 when most genes were downregulated (**Figure 7B**). The top 5 pathways with the most altered gene expression on D3 were the Interferon (mean: -3.81), Interleukin (mean:-3.33), TLR (mean: -2.93), Adaptive Immunity (mean: -2.89), and the Metabolism (mean: -1.88) pathways. However, most of the genes in the interferon pathway that were downregulated log_2_ ≥1.32 in Group 1 still had increased mRNA levels in Group 2 (**Figure 7B**). As was observed on D1, the downregulation of IL1RL1 was more pronounced in Group 2 and this was also true for the related gene IL1R2 (**Figure 7B**). Few genes (e.g., HLA-C, FCGRIA) were shared between pathways on D3 and those did not represent DEG.

### Association of Early Innate Responses With Vaccine-Induced Antibody Responses

To address the question whether innate gene expression signatures on D1 or D3 post vaccine prime could predict the magnitude and function of vaccine-elicited Env-specific antibody responses to vaccination, we tested for correlations between D1 or D3 DEG, additional genes with increased or decreased mRNA expression that were common to at least two top scoring pathways (see **Figure 7**) and elevated plasma cytokines with the magnitude of vaccine-induced 1086.c Env-specific plasma IgG responses, neutralizing antibodies, and antibody-dependent cytotoxicity function (15, 16). Time points were selected to represent peak vaccine-elicited adaptive immune responses after the initial three immunizations (week 14 or 2 weeks post the 3^rd^ immunization), vaccine-induced memory responses (week 20: 8 weeks post 3^rd^ immunization; week 32: 20 weeks post the 3^rd^ immunization and time of 4^th^ immunization), and week 34, the peak response to the late boost (2 weeks post the 4th and final immunization) (15, 16). Although our two vaccine regimens were primarily designed to enhance Env-specific antibodies with Fc-mediated effector function (15, 16), we also assessed the impact of D1 or D3 gene expression and plasma cytokines on vaccine-induced neutralizing antibodies. As the direction of changes in mRNA levels and plasma cytokines on D1 was similar in both groups (**Supplemental Table S2** and see **Figure 4**) and our group sizes were limited in number, we combined the data of Group 1 and Group 2 to test for biologically meaningful correlation between innate vaccine prime-induced signatures and vaccine-induced adaptive immune responses.

The mRNA expression of six DEG on D1 were positively correlated with plasma Env-specific IgG at week 14 and for four of these genes (C3AR1, TNFSF10, LILRA3, and IFITM1) a positive correlation was also seen at week 32 (**Table 2**). In contrast, higher HLA-DQB1 mRNA levels on D1 were associated with lower Env-specific plasma IgG concentrations at week 32. The magnitude of Tier 1 neutralizing antibodies at week 14, week 32, or week 34 could be associated with one, three, or four DEG respectively (**Table 3**). It should be noted that the mRNA expression levels of the D1 DEG LILRA3, IFITM1, IRF7, and DDIT3 were positively associated with Env-specific plasma IgG and neutralizing antibody responses and the shared pathway gene HLA-DQB1 negatively correlated with both responses (**Tables 2**, **3**). On D3, the higher mRNA levels of IL1RL1, the lower were Env-specific IgG and neutralizing antibody responses (**Tables 2**, **3**). Associations of innate immune parameters unique to Env-specific plasma IgG were the inverse correlation with IL6 mRNA on D1 and with MCP1 mRNA on D3. The D1 gene expression of C3AR1, TNFSF10, and CSF3R was positively associated with both Env-specific IgG and ADCC responses (**Tables 2**, **4**). Overall, the induction of 9 DEG on D1 was positively correlated with Env-specific IgG-mediated ADCC responses at week 34, two weeks after the late boost (**Table 4**). Similarly, D1 mRNA expression of the shared pathway genes IL2RA, CSF2RB, and FCGR1A were correlated with ADCC responses at week 34 (**Table 4**). Among the elevated plasma cytokines at D1, IL-6 was negatively associated plasma IgG at weeks 32 and 34. Although increased plasma cytokine levels on D1 did not appear to impact ADCC responses at week 34, IFNG mRNA levels were inversely correlated with ADCC responses at week 14 (**Table 4**), whereas the mRNA expression of CCL2, the gene encoding MCP-1, were positively correlated with ADCC at week 34 (**Table 4**). On D3, few associations between mRNA expression and antibody responses were noted. Furthermore, with the exception of BCL2L1, these associations represented inverse correlations (**Tables 2**–**4**). In particular, the lower IL18 mRNA expression was, the lower were Env-specific plasma IgG, ADCC, and neutralizing antibody responses (**Tables 2**–**4**).

**Table 2:** Correlation of early gene expression with vaccine-induced Env-specific IgG.

Parameter	Spearman Correlation											
	week 14			week 20			week 32			week 34		
	r	p	q	r	p	q	r	p	q	r	p	q
**D1 DEG**												
SERPING1	0.3464	0.2061	0.6314	0.0250	0.9336	0.9682	0.4536	0.0915	0.5484	0.3393	0.2161	0.6415
**C3AR1**	**0.5321**	**0.0438**	**0.5158**	0.3607	0.1870	0.6212	**0.5964**	**0.0213**	**0.5158**	0.3821	0.1607	0.6147
IL1RN	0.4036	0.1370	0.5932	0.1500	0.5934	0.8379	0.4286	0.1127	0.5771	0.3714	0.1735	0.6182
TNFAIP6	0.4679	0.0808	0.5470	0.1893	0.4983	0.7907	0.3607	0.1870	0.6212	0.4643	0.0834	0.5470
PLAUR	-0.0750	0.7925	0.9346	-0.1857	0.5067	0.7907	-0.1357	0.6297	0.8695	-0.0107	0.9744	0.9907
FPR2	0.5071	0.0562	0.5158	0.2107	0.4498	0.7820	0.4071	0.1334	0.5900	0.4464	0.0972	0.5625
SOCS3	0.4893	0.0666	0.5318	0.2571	0.3538	0.7378	0.5000	0.0602	0.5158	0.3107	0.2592	0.6848
OAS1	0.3571	0.1917	0.6278	0.0821	0.7728	0.9263	0.3071	0.2649	0.6973	0.3607	0.1870	0.6213
***TNFSF10***	0.4929	0.0644	0.5207	0.3000	0.2767	0.6999	***0.6786***	***0.0068***	***0*.4927**	0.4071	0.1334	0.5900
CCR1	0.4000	0.1408	0.5999	0.0143	0.9642	0.9833	0.2107	0.4498	0.7820	0.4357	0.1063	0.5737
**LILRA3**	**0.6036**	**0.0195**	**0.5158**	0.3500	0.2012	0.6314	**0.5214**	**0.0488**	**0.5158**	**0.5393**	**0.0406**	**0.5158**
**IFITM1**	**0.5321**	**0.0438**	**0.5158**	0.2536	0.3607	0.7379	**0.6036**	**0.0195**	**0.5158**	0.4607	0.0861	0.5470
ARG2	0.4821	0.0711	0.5358	0.1821	0.5150	0.7960	0.3929	0.1485	0.6004	0.3964	0.1446	0.6003
CD82	0.4179	0.1227	0.5900	0.2107	0.4498	0.7820	0.3464	0.2061	0.6313	0.2321	0.4039	0.7633
**IRF7**	**0.5607**	**0.0322**	**0.5158**	0.2500	0.3677	0.7433	0.4429	0.1002	0.5650	0.5107	0.0543	0.5158
IFI35	0.2607	0.3469	0.7378	-0.2250	0.4189	0.7633	0.0357	0.9031	0.9557	0.2964	0.2827	0.6999
NFKBIA	0.4429	0.1002	0.5650	0.1571	0.5756	0.8341	0.3750	0.1692	0.6169	0.3571	0.1917	0.6277
**CSF3R**	**0.5500**	**0.0362**	**0.5158**	0.2250	0.4189	0.7633	0.4357	0.1063	0.5737	0.5071	0.0562	0.5158
**DDIT3**	**0.5214**	**0.0488**	**0.5158**	0.2036	0.4657	0.7881	0.3000	0.2767	0.6999	**0.5607**	**0.0322**	**0.5158**
GP1BB	0.2036	0.4657	0.7881	0.1143	0.6858	0.8988	0.1500	0.5934	0.8379	0.2607	0.3469	0.7378
CLU	0.0786	0.7827	0.9263	0.0357	0.9031	0.9557	0.1857	0.5067	0.7907	0.0464	0.8726	0.9547
TNFAIP3	0.5107	0.0543	0.5158	0.2036	0.4657	0.7810	0.3964	0.1446	0.6003	0.4107	0.1297	0.5900
IL1RL1	0.3643	0.1824	0.6212	-0.0750	0.7925	0.9346	0.3107	0.2592	0.6848	0.4786	0.0735	0.5358
HLA-DMA	-0.2607	0.3469	0.7378	0.1000	0.7208	0.9098	-0.2214	0.4266	0.7633	-0.2143	0.4407	0.7785
KIT	-0.1000	0.7241	0.9098	-0.3214	0.2424	0.6804	-0.1464	0.6024	0.8473	0.1000	0.7241	0.9098
**D1 Shared Pathway Genes** ^c^												
IL2RA	0.3429	0.2111	0.6408	0.1179	0.6763	0.8933	0.2357	0.3966	0.7633	0.2179	0.4342	0.7750
CSF2RB	0.4821	0.0711	0.5358	0.2214	0.4266	0.7633	0.5036	0.0582	0.5158	0.4071	0.1333	0.5900
FCGR1A	0.2071	0.4578	0.7876	0.2821	0.3074	0.7138	0.2893	0.2949	0.7046	0.1857	0.5067	0.7907
DUSP4	-0.0464	0.8764	0.9547	0.1429	0.6115	0.8548	0.1821	0.5151	0.7960	-0.0786	0.7827	0.9262
**HLA-DQB1**	**-0.5714**	**0.0286**	**0.5158**	-0.3464	0.2061	0.6314	**-0.6036**	**0.0195**	**0.5158**	-0.4107	0.1297	0.5900
HLA-DRB1	0.1000	0.7241	0.9098	0.1071	0.7041	0.9060	0.2393	0.3892	0.7592	0.0393	0.8929	0.9547
**D1 Cytokine Genes** ^d^												
IFNA2	-0.1857	0.5067	0.7906	-0.2714	0.3269	0.7335	-0.2429	0.3820	0.7548	-0.0036	0.9948	0.9999
IL6	-0.0321	0.9132	0.9558	-0.0857	0.7630	0.9220	-0.0250	0.9336	0.9682	0.0321	0.9132	0.9557
IL18	0.1964	0.4819	0.7881	-0.1036	0.7144	0.9098	0.2321	0.4039	0.7633	0.1786	0.5235	0.7960
IFNG	-0.3536	0.1964	0.6314	-0.3250	0.2370	0.6739	-0.1857	0.5067	0.7907	-0.2750	0.3203	0.7310
MCP1	0.0786	0.7827	0.9262	-0.0786	0.7827	0.9262	-0.1179	0.6763	0.8933	0.1179	0.6763	0.8933
**D1 Cytokine Proteins**												
IFN-α	-0.3750	0.1692	0.6169	-0.1214	0.6669	0.8932	-0.3464	0.2061	0.6314	-0.2571	0.3538	0.7378
**IL-6**	-0.4540	0.0905	0.5470	-0.3843	0.1573	0.6147	**-0.5290**	**0.0447**	**0.5158**	**-0.5827**	**0.0248**	**0.5158**
IL-18	-0.2643	0.3402	0.7378	-0.2643	0.3402	0.7378	-0.3107	0.2592	0.6848	-0.2393	0.3892	0.7592
IFN-γ	-0.2073	0.4553	0.7873	-0.2288	0.4090	0.7633	-0.0447	0.8753	0.9547	-0.0840	0.7654	0.9220
MCP-1	-0.2321	0.4039	0.7633	-0.3214	0.2425	0.6804	0.0036	0.9948	0.1000	-0.4786	0.0734	0.5358
IL-1RA	0.1786	0.5235	0.7960	-0.1607	0.5667	0.8284	-0.0429	0.8828	0.9547	0.1214	0.6669	0.8932
**D3 DEG**												
CLU	-0.2396	0.4086	0.7633	0.0418	0.8915	0.9547	-0.4198	0.1368	0.5932	-0.2263	0.4356	0.7753
GP1BB	-0.2835	0.3253	0.7335	0.1165	0.6930	0.9029	-0.4286	0.1281	0.5900	-0.2440	0.3998	0.7633
**BCL2L1**	-0.0637	0.8319	0.9451	**0.5472**	**0.0458**	**0.5158**	0.2659	0.3573	0.7378	-0.1472	0.6158	0.8590
**IL1RL1**	-0.5297	0.0544	0.5158	-0.1956	0.5022	0.7907	-0.1165	0.6930	0.9029	**-0.6483**	**0.0144**	**0.5158**
ILR2	-0.2967	0.3026	0.7047	-0.2483	0.3911	0.7606	-0.0549	0.8557	0.9506	-0.4374	0.1198	0.5900
**D3 Shared Pathway Genes**												
HLA-C	-0.1253	0.6706	0.8933	-0.2615	0.3656	0.7433	-0.2747	0.3411	0.7378	0.2396	0.4086	0.7633
FCGR1A	-0.2527	0.3825	0.7548	-0.0637	0.8319	0.9451	0.1033	0.7270	0.9101	-0.2703	0.3492	0.7378
**D3 Cytokine Genes**												
IFNA2	-0.4330	0.1239	0.5900	-0.4154	0.1412	0.5999	0.0242	0.9396	0.9729	-0.3670	0.1973	0.6314
IL6	-0.2044	0.4827	0.7881	-0.0769	0.7965	0.9360	0.0593	0.8438	0.9484	-0.2044	0.4827	0.7881
**IL18**	**-0.7231**	**0.0047**	**0.4927**	-0.4725	0.0905	0.5470	-0.2703	0.3492	0.7378	-**0.6879**	**0.0082**	**0.4927**
IFNG	-0.3978	0.1602	0.6147	-0.1516	0.6051	0.8494	-0.0461	0.8795	0.9547	-0.4725	0.0905	0.5470
**MCP1**	-0.5516	0.0438	0.5158	-0.2747	0.3411	0.7378	-0.2176	0.4541	0.7873	-**0.6000**	**0.0261**	**0.5158**
IL1RN	-0.3714	0.1918	0.6277	0.1121	0.7043	0.9060	-0.0989	0.7385	0.9136.	-0.2352	0.4175	0.7633
**D3 Cytokine Proteins**												
IFN-α	-0.0632	0.8382	0.9484	0.1348	0.6589	0.8896	0.3026	0.3121	0.7212	0.2944	0.3264	0.7335
IL-6	0.3232	0.2949	0.7046	-0.0961	0.7564	0.9211	0.0874	0.7820	0.9263	0.3319	0.2820	0.6999
IL-18	-0.0660	0.8231	0.9451	0.2552	0.3757	0.7526	0.1694	0.5597	0.8284	-0.1012	0.7298	0.9117
IFN-γ	-0.3160	0.2689	0.6988	-0.0022	0.9968	0.1000	0.3005	0.2870	0.7021	-0.0354	0.9062	0.9557
**MCP-1**	**-0.7890**	**0.0013**	**0.4228**	-0.2835	0.3253	0.7335	0.2527	0.3825	0.7548	-0.5165	0.0615	0.5158
IL-1RA	0.0399	0.8967	0.9556	-0.0446	0.8839	0.9547	0.1948	0.5030	0.7907	0.1338	0.6483	0.8824

a bold font corresponds to p<0.05.

b bold and italic font corresponds to p<0.01

c Genes shared between the top five scoring pathways on D1 also included IL1RN, SOCS3, IFITM1, IRF7, NFKBIA, and KIT, genes that are included in the DEG.

d The gene encoding IL-1RA is ILRN that is included in the DEG.

**Table 3:** Correlation of early gene expression with vaccine-induced Env-specific neutralizing antibodies.

Parameter	Spearman Correlation								
	week 14			week 32			week 34		
	r	p	q	r	p	q	r	p	q
**D1 DEG**									
SERPING1	0.1500	0.5934	0.8379	0.2000	0.4738	0.7881	0.2679	0.3334	0.7378
C3AR1	0.3107	0.2592	0.6848	0.3786	0.1649	0.6147	0.3464	0.2061	0.6313
IL1RN	0.2393	0.3892	0.7592	0.3250	0.2370	0.6739	0.4393	0.1032	0.5737
**TNFAIP6**	0.3214	0.2425	0.6804	0.3607	0.1870	0.6212	**0.5214**	**0.0488**	**0.5158**
PLAUR	-0.4750	0.0759	0.5415	-0.5143	0.0524	0.5158	-0.1929	0.4901	0.7907
FPR2	0.1429	0.6115	0.8548	0.1857	0.5067	0.7907	0.3714	0.1735	0.6182
**SOCS3**	0.4821	0.0711	0.5358	**0.5357**	**0.0422**	**0.5158**	0.4571	0.0888	0.5470
OAS1	0.4179	0.1227	0.7907	0.1929	0.4901	0.7378	-0.3714	0.1735	0.5900
TNFSF10	0.2893	0.2949	0.7046	0.4107	0.1297	0.5900	0.3893	0.1525	0.6055
CCR1	0.0179	0.9540	0.9803	0.0714	0.8025	0.9365	0.4679	0.0808	0.5470
**LILRA3**	0.4071	0.1334	0.5900	0.4500	0.0944	0.5506	**0.5357**	**0.0422**	**0.5158**
**IFITM1**	**0.5964**	**0.0213**	**0.5158**	**0.6429**	**0.0116**	**0.4927**	0.5000	0.0602	0.5158
ARG2	0.3393	0.2161	0.6415	0.3750	0.1692	0.6169	0.4179	0.1227	0.5900
CD82	0.2607	0.3469	0.7378	0.3107	0.2592	0.6848	0.2929	0.2888	0.7021
**IRF7**	0.3929	0.1485	0.6004	0.4429	0.1002	0.5650	**0.5679**	**0.0297**	**0.5158**
IFI35	-0.1964	0.4819	0.7881	-0.2500	0.3678	0.7433	0.1607	0.5667	0.8284
NFKBIA	0.1893	0.4983	0.7907	0.2571	0.3538	0.7378	0.3607	0.1870	0.6212
CSF3R	0.3393	0.2160	0.6415	0.3821	0.1607	0.6147	0.5179	0.0506	0.5158
**DDIT3**	0.2857	0.3012	0.7047	0.2964	0.2827	0.6999	**0.6464**	**0.0110**	**0.4927**
GP1BB	0.0321	0.9132	0.9557	0.0536	0.8525	0.9506	0.2071	0.4578	0.7876
CLU	-0.2214	0.4266	0.7633	-0.1964	0.4819	0.7880	-0.1929	0.4901	0.7907
TNFAIP3	0.1893	0.4983	0.8824	0.1286	0.6482	0.7907	0.4714	0.0783	0.5470
IL1RL1	-0.0964	0.7337	0.9117	-0.1536	0.5844	0.8379	0.1893	0.4983	0.7907
HLA-DMA	-0.4286	0.1127	0.5771	-0.3786	0.1649	0.6147	-0.2536	0.3607	0.7379
**KIT**	-0.4786	0.0735	0.5358	**-0.5464**	**0.0376**	**0.5158**	-0.2214	0.4266	0.7633
**D1 Shared Pathway Genes** ^c^									
IL2RA	0.0893	0.7532	0.9189	0.1250	0.6575	0.8895	0.1214	0.6669	0.8932
CSF2RB	0.2214	0.4266	0.7633	0.3286	0.2317	0.6722	0.4071	0.1334	0.5900
FCGR1A	0.2393	0.3892	0.7592	0.3179	0.2479	0.6804	0.3643	0.1824	0.6212
DUSP4	-0.3429	0.2111	0.6408	-0.2214	0.4266	0.7633	-0.1607	0.5667	0.8284
***HLA-DQB1***	***-0.6857***	***0.0061***	***0.4927***	***-0.6964***	***0.0051***	***0.4927***	-0.4571	0.0888	0.5470
HLA-DRB1	0.0714	0.8025	0.9365	0.1500	0.5934	0.8379	-0.0070	0.9847	0.9980
**D1 Cytokine Genes** ^d^									
IFNA2	-0.3786	0.1649	0.6147	-0.4321	0.1094	0.5737	-0.1500	0.5934	0.8379
IL6	-0.4321	0.1094	0.5737	-0.3964	0.1446	0.6003	-0.1000	0.7241	0.9098
IL18	-0.3893	0.1525	0.6055	-0.4607	0.0861	0.5470	-0.2286	0.4114	0.7633
IFNG	-0.4607	0.0861	0.5470	-0.5071	0.0562	0.5158	-0.5000	0.0602	0.5158
MCP1	-0.2321	0.4039	0.7633	-0.2107	0.4498	0.7820	0.2250	0.4190	0.7633
**D1 Cytokine Proteins**									
IFN-α	-0.1321	0.6389	0.8785	-0.0464	0.8726	0.9547	-0.2036	0.4657	0.7880
IL-6	0.0643	0.8196	0.9557	0.0304	0.9158	0.9450	-0.2574	0.3514	0.7378
IL-18	-0.1250	0.6575	0.8898	-0.0429	0.8828	0.9547	-0.2679	0.3334	0.7378
IFN-γ	0.3378	0.2170	0.6415	0.4004	0.1394	0.5996	0.0840	0.7654	0.9220
MCP-1	-0.0321	0.9132	0.9557	0.1786	0.5235	0.7960	-0.3500	0.2012	0.6314
IL-1RA	-0.2000	0.4738	0.7880	-0.2036	0.4657	0.7880	-0.0786	0.7827	0.9263
**D3 DEG**									
CLU	-0.3978	0.1602	0.6147	-0.1780	0.5423	0.8141	-0.0901	0.7616	0.9220
GP1BB	-0.1736	0.5526	0.8239	0.0945	0.7500	0.9189	0.0154	0.9637	0.9833
BCL2L1	0.0769	0.7965	0.9360	0.4330	0.1239	0.5900	-0.0153	0.9637	0.9833
***IL1RL1***	-0.0374	0.9035	0.9557	0.0637	0.8319	0.9451	***-0.6835***	***0.0088***	***0.4927***
**ILR2**	-0.3275	0.2530	0.6848	-0.2088	0.4731	0.7881	**-0.6132**	**0.0224**	**0.5158**
**D3 Shared Pathway Genes**									
HLA-C	0.0154	0.9638	0.9833	-0.0901	0.7616	0.9220	0.4681	0.0938	0.5506
FCGR1A	-0.4110	0.1458	0.6003	-0.3802	0.1808	0.6212	-0.3407	0.2335	0.6724
**D3 Cytokine Genes**									
IFNA2	-0.1165	0.6930	0.9029	-0.3099	0.2806.	0.6999	-0.5297	0.0543	0.5158
IL6	-0.1121	0.7042	0.9060	-0.2615	0.3656	0.7433	-0.1692	0.5629	0.8284
***IL18***	-0.3011	0.2951	0.7046	-0.3319	0.2464	0.6804	-***0.8242***	***0.0005***	***0.3471***
IFNG	0.0418	0.8915	0.9547	-0.0505	0.8676	0.9547	-0.4286	0.1281	0.5900
MCP1	-0.0549	0.8557	0.9506	-0.0022	1.0000	1.0000	-0.4945	0.0750	0.5411
IL1RN	-0.2307	0.4265	0.7633	-0.0725	0.8083	0.9400	-0.0417	0.8915	0.9547
**D3 Cytokine Proteins**									
IFN-α	0.3439	0.2484	0.6804	0.4044	0.1706	0.6181	0.4237	0.1494	0.6005
IL-6	0.2096	0.5000	0.7907	-0.0262	0.9487	0.9764	0.0262	0.9487	0.9764
IL-18	-0.1848	0.5243	0.7960	0.0726	0.8049	0.9377	-0.1210	0.6784	0.8944
IFN-g	-0.2077	0.4732	0.7881	-0.0530	0.8581	0.9517	-0.0972	0.7402	0.9136
MCP-1	-0.1077	0.7156	0.9098	-0.0066	0.9879	0.9983	-0.3319	0.2464	0.6804
IL-1RA	-0.5328	0.0532	0.5158	-0.4811	0.0843	0.5470	0.0023	1.0000	1.0000

a bold font corresponds to p < 0.05.

b bold and italic font corresponds to p < 0.01.

c Genes shared between the top five scoring pathways on D1 also included IL1RN, SOCS3, IFITM1, IRF7, and NFKBIA, genes that are included in the DEG.

d The gene encoding IL-1RA is ILRN that is included in the DEG.

**Table 4:** Correlation of early gene expression with vaccine-induced Env-specific ADCC.

Parameter		Spearman Correlation															
		week 14						week 20				week 32			week 34		
		r	p	q	r	p	q	r	p	q	r	p	q
**D1 DEG**	
SERPING1		-0.0464		0.8726		0.9547		0.0964		0.7337	0.9117	0.2679	0.3334	0.7378	0.4684	0.0799	0.5470
**C3AR1** ^a^		-0.0893		0.7532		0.9189		0.3929		0.1485	0.6004	0.2964	0.2827	0.6999	**0.6363**	**0.0129**	**0.5121**
***IL1RN*** ^b^		-0.0071		0.9847		0.9981		0.3179		0.2479	0.6804	0.4321	0.1094	0.5737	***0.6768***	***0.0073***	***0.4927***
TNFAIP6		0.1571		0.5756		0.8342		0.1214		0.6669	0.8932	0.1393	0.6205	0.8621	0.5016	0.0590	0.5158
PLAUR		-0.3714		0.1735		0.6182		0.0607		0.8324	0.9450	0.0214	0.9438	0.9758	0.3689	0.1751	0.6184
FPR2		0.1071		0.7049		0.9060		0.1143		0.6858	0.8988	0.1786	0.5253	0.7960	0.3947	0.1453	0.6003
***SOCS3***		0.3000		0.2767		0.6999		**0.5429**		**0.0391**	**0.5158**	***0.7393***	***0.0023***	***0.4664***	**0.5275**	**0.0458**	**0.5158**
OAS1		0.1286		0.6482		0.8824		0.0357		0.9031	0.9557	0.1571	0.5756	0.8342	0.4316	0.1091	0.5737
**TNFSF10**		-0.0393		0.8929		0.9547		0.3679		0.1779	0.6212	0.4571	0.0888	0.5470	**0.5699**	**0.0292**	**0.5158**
**CCR1**		0.2857		0.3012		0.7047		0.2143		0.4421	0.7785	0.3500	0.2012.	0.6314	**0.6436**	**0.0117**	**0.4927**
LILRA3		0.2000		0.4738		0.7881		0.2929		0.2888	0.7021	0.2786	0.3139	0.7212	0.4980	0.0611	0.5158
IFITM1		0.2750		0.3203		0.7310		0.2286.		0.4114	0.7633	0.3393	0.2161	0.6415	0.4832	0.0700	0.5358
ARG2		0.2679		0.3335		0.7378		0.1500		0.5934	0.8379	0.2571	0.3538	0.7378	0.3523	0.1964	0.6314
CD82		0.1750		0.5320		0.8022		0.1607		0.5667	0.8284	0.2857	0.3012	0.7047	0.3043	0.2673	0.6979
**IRF7**		0.2929		0.2888		0.7021		0.2214		0.4266	0.7633	0.3286	0.2317	0.6722	**0.5662**	**0.0304**	**0.5158**
IFI35		0.2250		0.4189		0.7633		0.0607		0.8324	0.9451	0.2250	0.4189	0.7633	0.4057	0.1336	0.5900
NFKBIA		0.1393		0.6205		0.8621		0.0571		0.8425	0.9484	0.1929	0.4901	0.7907	0.4131	0.1263	0.5900
**CSF3R**		0.2786		0.3139		0.7212		0.1643		0.5580	0.8283	0.2643	0.3401	0.7378	**0.5348**	**0.0425**	**0.5158**
**DDIT3**		0.0893		0.7532		0.9189		0.1786		0.5253	0.7960	0.1286	0.6482	0.8824	**0.5275**	**0.0458**	**0.5158**
GP1BB		0.0036		0.9949		0.9999		-0.2464		0.3748	0.7526	-0.2929	0.2888	0.7021	0.0664	0.0814	0.9444
CLU		-0.0786		0.7827		0.9263		-0.3286		0.2317	0.6723	-0.1750	0.5320	0.8022	-0.0646	0.8187	0.9451
***TNFAIP3***		-0.0250		0.9336		0.9682		0.3500		0.2012	0.6314	0.4286	0.1127	0.5771	***0.6584***	***0.0096***	***0.4927***
IL1RL1		-0.0536		0.8505		0.9506		-0.1357		0.6297	0.8695	-0.1179	0.6763	0.8933	0.4186	0.1209	0.5900
**HLA-DMA**		-0.5071		0.0562		0.5158		-0.3143		0.2536	0.6848	**-0.5607**	**0.0322**	**0.5158**	-0.3025	0.2703	0.6988
KIT		-0.3250		0.2370		0.6739		-0.2143		0.4421	0.7785	-0.3036	0.2708	0.6988	0.1778	0.5239	0.7960
**D1 Shared Pathway Genes** ^c^																	
**IL2RA**	0.3357		0.2212		0.6510		0.4571		0.0888		0.5470	**0.5607**	**0.0322**	**0.5158**	**0.5496**	**0.0364**	**0.5158**
**CSF2RB**	0.1286		0.6482		0.8824		0.2964		0.2827		0.6999	0.4321	0.1094	0.5737	**0.5939**	**0.0221**	**0.5158**
**FCGR1A**	-0.1964		0.4819		0.7880		**0.5571**		**0.0335**		**0.5158**	0.3393	0.2161	0.6414	**0.5625**	**0.0317**	**0.5158**
DUSP4	-0.4714		0.0783		0.5470		0.2036		0.4657		0.7881	0.3107	0.2592	0.6848	0.3799	0.1618	0.6147
HLA-DQB1	-0.4964		0.0623		0.5158		-0.3179		0.2479		0.6804	-0.4357	0.1063	0.5737	-0.3596	0.1867	0.6212
HLA-DRB1	-0.2429		0.3820		0.7548		-0.1500		0.5934		0.8379	-0.3000	0.2767	0.6999	-0.0922	0.7421	0.9136
**D1 Cytokine Genes** ^d^																	
IFNA2	-0.1678		0.5492		0.8226		0.0536		0.8525		0.9506	0.0856	0.7630	0.9220	0.2545	0.3561	0.7378
IL6	-0.4036		0.1370		0.5931		-0.0250		0.9336		0.9682	-0.0321	0.9132	0.9557	0.3043	0.2673	0.6979
IL18	-0.4214		0.1193		0.5900		-0.2250		0.4189		0.7633	-0.1036	0.7144	0.9098	0.2102	0.4478	0.7820
**IFNG**	**-0.5500**		**0.0362**		**0.5158**		-0.2643		0.3402		0.7378	-0.1643	0.5579	0.8283	-0.0387	0.8917	0.9550
***MCP1***	-0.1500		0.5934		0.8379		0.5179		0.0506		0.5158	0.3786	0.1649	0.6147	***0.6621***	***0.0091***	***0.4927***
**D1 Cytokine Proteins**																	
IFN-α	-0.0714		0.8025		0.9365		-0.0714		0.8025		0.9365	-0.3536	0.1964	0.6313	0.0590	0.8342	0.9455
IL-6	0.1144		0.6829		0.8984		-0.1001		0.7212		0.9098	-0.0536	0.8497	0.9506	-0.3840	0.1567	0.6147
IL-18	0.2214		0.4266		0.7633		-0.0893		0.7532		0.9188	-0.2214	0.4266	0.7633	0.0387	0.8917	0.9547
IFN-γ	0.1001		0.7213		0.9098		-0.0572		0.8396		0.9484	-0.0947	0.7361	0.9130	0.0406	0.8849	0.9547
MCP-1	0.1036		0.7144		0.9098		-0.0786		0.7827		0.9262	0.2536	0.3607	0.7379	-0.1070	0.7021	0.9060
IL-1RA	0.1964		0.4819		0.7881		-0.1500		0.5934		0.8379	-0.1321	0.6389	0.8785	0.1180	0.6726	0.8933
**D3 DEG**																	
**CLU**	0.1121		0.7043		0.9036		-0.3275		0.2530		0.6906	**-0.5736**	**0.0349**	**0.5243**	-0.2978	0.2981	0.7089
GP1BB	0.0549		0.8557		0.9504		-0.1692		0.5629		0.8312	-0.5033	0.0694	0.5446	-0.2556	0.3742	0.7604
BCL2L1	-0.2528		0.3825		0.7650		-0.0330		0.9155		0.9565	-0.1429	0.6266	0.8651	-0.0133	0.9663	0.9836
IL1RL1	-0.3143		0.2735		0.7036		-0.1560		0.5944		0.8376	0.1253	0.6706	0.8904	-0.3911	0.1663	0.9196
ILR2	-0.1604		0.5838		0.8376		-0.2967		0.3025		0.7089	0.0637	0.8319	0.9446	-0.3400	0.2324	0.6774
**D3 Shared Pathway Genes**																	
HLA-C	0.1033		0.7270		0.9115		0.1868		0.5221		0.7999	-0.0637	0.8319	0.9446	0.1978	0.4942	0.7962
FCGR1A	-0.2351		0.4174		0.7633		-0.1076		0.7156		0.9098	0.2307	0.4265	0.7633	0.0422	0.8870	0.9547
**D3 Cytokine Genes**																	
IFNA2	-0.3802		0.1808		0.6282		-0.5209		0.0591		0.5243	0.0066	0.9879	0.9982	-0.4422	0.1144	0.5820
IL6	-0.2835		0.3253		0.7382		-0.2088		0.4731		0.7933	-0.0637	0.8319	0.9446	-0.3022	0.2907	0.7081
***IL18***	-0.6352		0.0171		0.5243		-0.2659		0.3573		0.7453	***-0.7534***	***0.0028***	***0.4741***	***-0.7231***	***0.0047***	***0.5001***
IFNG	-0.3758		0.1862		0.6282		-0.1604		0.5838		0.8376	0.2044	0.4827	0.7933	-0.5111	0.0641	0.5292
**MCP1**	-0.2044		0.4827		0.7933		0.2967		0.3025		0.7089	**0.6176**	**0.0212**	**0.5243**	0.0333	0.9114	0.9565
IL1RN	-0.1780		0.5423		0.8183		0.0593		0.8438		0.9480	0.1912	0.5121	0.7999	-0.0022	0.9970	0.9999
**D3 Cytokine Proteins**																	
IFN-α	-0.2063		0.4958		0.7962		0.1183		0.6991		0.9036	0.1898	0.5316	0.8063	0.1936	0.5224	0.7999
IL-6	0.1922		0.5513		0.8281		-0.0437		0.8974		0.9563	-0.0874	0.7821	0.9235	-0.0133	0.9615	0.9830
IL-18	-0.0968		0.7413		0.9152		-0.1298		0.6562		0.8862	0.0484	0.8707	0.9552	-0.0645	0.8521	0.9446
IFN-γ	-0.3624		0.2021		0.6388		-0.1658		0.5684		0.8318	0.0685	0.8159	0.9432	0.0391	0.8935	0.9552
**MCP-1**	**-0.5692**		**0.0366**		**0.5243**		-0.2659		0.3573		0.7453	-0.0462	0.8796	0.9552	-0.3845	0.1741	0.6217
IL-1RA	0.1009		0.7327		0.9131		-0.2229		0.4417		0.7872	-0.0211	0.9482	0.9759	0.0427	0.8856	0.9552

a bold font corresponds to p < 0.05.

b bold and italic font corresponds to p < 0.01.

c Genes shared between the top five scoring pathways on D1 also included IL1RN, SOCS3, IFITM1, IRF7, NFKBIA, and KIT, genes that are included in the DEG.

d The gene encoding IL-1RA is ILRN that is included in the DEG.

Based on these findings, we assessed whether vaccine prime-induced responses also correlated with B cell populations as antibody producing cells. The mRNA levels of SOCS3 were positively and the mRNA levels of HLA-DMA were negatively correlated with total CD27^+^ memory B cells in peripheral blood at week 34, but not with lymph node memory B cells (**Table 5**). However, there was a positive correlation of six DEG with lymph node CXCR5^+^ germinal center (GC) B cell frequencies at week 34 (**Table 5**; see **Supplemental Figure S1**). The latter were also associated with IL6 mRNA levels, but not with IL-6 plasma concentrations on D1 (**Table 5**). There was no overlap between the genes that correlated with peripheral blood memory B cells or with lymph node GC B cells. Follicular T helper cells (T_FH_) provide critical signals for B cell activation and antibody maturation in lymph nodes (23–26), and, in turn, the T_FH_ responses are directly dependent on the priming by antigen presenting cells and the local immune milieu in lymph nodes (27–30). Env-specific lymph node T_FH_ frequencies (see **Supplemental Figure S2**) were correlated with mRNA levels of 19 of the 25 DEG with increased mRNA on D1, one shared pathway gene (CSF2RB), and with IL6 and CCL2 (MCP1) mRNA levels (**Table 5**). The frequencies of Env-specific lymph node T_FH_ were only weakly associated with GC B cells (r=0.4941, p=0.0540); the caveat being that these B cells were not HIV Env-specific but represented total GC B cells. Note that Env-specific lymph node T_FH_ frequencies were positively correlated with OX40^+^CD137^+^ T_FH_ frequencies (r=0.5956, p=0.0274) after *in vitro* SEB stimulation. SEB-activated OX40^+^CD137^+^ T_FH_ frequencies were also weakly correlated with GC B cells (r=0.5235, p=0.0567) and showed a positive correlation with lymph node CD27^+^ memory B cells (r=0.7363, p=0.0037). Combined, these data support the idea that the measurement of total GC B cells was likely representative of Env-specific GC B cells in our study. B cell frequencies or Env-specific lymph node T_FH_ frequencies were not associated with D3 cytokines or gene signatures.

**Table 5:** Correlation of early gene expression with follicular T helper cells (T_FH_) or with memory or germinal center (GC) B cells (week 34).

Parameter	Spearman Correlation											
	PBMC			Lymph Nodes								
	Memory B cells			Memory B cells			GC B cells			Env-specific T_FH_		
	r	p	q	r	p	q	r	p	q	r	p	q
**D1 DEG**												
***SERPING1*** *^a^* ^,b^	0.3914	0.1492	0.7379	0.1464	0.6024	0.8743	**0.6214**	**0.0155**	**0.3200**	***0.6536***	***0.0099***	***0.3200***
**C3AR1**	0.1487	0.5209	0.8529	0.1036	0.7144	0.9287	0.3393	0.2161	0.7714	**0.5429**	**0.0391**	**0.4579**
**IL1RN**	0.3968	0.1432	0.7379	0.2679	0.3334	0.7868	**0.5286**	**0.0454**	**0.4825**	**0.7286**	**0.0029**	**0.3153**
**TNFAIP6**	0.1877	0.5002	0.8523	0.2821	0.3074	0.7861	0.4107	0.1297	0.7202	***0.7143***	***0.0037***	***0.3153***
**PLAUR**	0.2735	0.3216	0.7861	0.1571	0.5756	0.8658	**0.6286**	**0.0141**	**0.3200**	**0.5393**	**0.0406**	**0.4578**
**FPR2**	0.2413	0.3835	0.8163	0.3107	0.2592	0.7714	0.3643	0.1824	0.7458	**0.6500**	**0.0105**	**0.3200**
**SOCS3**	**0.5594**	**0.0324**	**0.4578**	0.3429	0.2111	0.7714	0.3107	0.2592	0.7714	0.3857	0.1566	0.7379
***OAS1***	0.3021	0.2719	0.7729	0.2929	0.2888	0.7729	0.4357	0.1063	0.6703	***0.7643***	***0.0014***	***0.3153***
**TNFSF10**	0.4004	0.1394	0.7378	0.2964	0.2827	0.7729	0.4000	0.1408	0.7378	**0.5679**	**0.0297**	**0.4426**
**CCR1**	0.2520	0.3622	0.8163	0.3750	0.1692	0.7379	0.4214	0.1193	0.6857	**0.6250**	**0.0148**	**0.3200**
**LILRA3**	0.1859	0.5041	0.8523	0.3571	0.1917	0.7667	0.3107	0.2592	0.7714	**0.5429**	**0.0391**	**0.4578**
IFITM1	0.1680	0.5466	0.8543	0.0143	0.9642	0.9907	0.1714	0.5406	0.8529	0.3500	0.2012	0.7714
ARG2	0.2806	0.3087	0.7861	0.2464	0.3748	0.8163	0.3107	0.2592	0.7714	**0.5393**	**0.0462**	**0.4578**
**CD82**	0.3682	0.1763	0.7379	0.2714	0.3269	0.7861	0.3250	0.2370	0.7714	**0.6214**	**0.0155**	**0.3200**
**IRF7**	0.1930	0.4875	0.8512	0.2321	0.4039	0.8167	0.2750	0.3203	0.7861	**0.5714**	**0.0286**	**0.4426**
**IFI35**	0.3682	0.1763	0.7379	0.2143	0.4421	0.8367	**0.5393**	**0.0406**	**0.4578**	**0.5250**	**0.0471**	**0.4864**
***NFKBIA***	0.2520	0.3621	0.8163	0.1964	0.4819	0.8512	0.3679	0.1779	0.7379	***0.6893***	***0.0057***	***0.3166***
**CSF3R**	0.1180	0.6735	0.9054	0.2393	0.3892	0.8163	0.3393	0.2161	0.7714	**0.5500**	**0.0362**	**0.4578**
***DDIT3***	0.1662	0.5512	0.8544	0.2714	0.3268	0.7861	0.3321	0.2264	0.7714	***0.7071***	***0.0042***	***0.3153***
***GP1BB***	0.1019	0.7167	0.9287	0.3071	0.2649	0.7729	0.3536	0.1964	0.7714	***0.6571***	***0.0094***	***0.3200***
CLU	0.1948	0.4837	0.8512	0.1750	0.5320	0.8529	0.4214	0.1193	0.6857	0.4500	0.0944	0.6415
***TNFAIP3***	0.3342	0.2221	0.7714	0.2857	0.3012	0.7861	0.4536	0.0915	0.6415	***0.7214***	***0.0033***	***0.3135***
***IL1RL1***	-0.0858	0.7602	0.9302	0.0500	0.8626	0.9550	***0.6786***	***0.0068***	***0.3166***	0.4607	0.0861	0.6415
**HLA-DMA**	**-0.5719**	**0.0281**	**0.4426**	-0.0179	0.9540	0.9907	-0.3036	0.2708	0.7729	-0.0929	0.7435	0.9202
**KIT**	0.0393	0.8901	0.9709	0.0607	0.8324	0.9483	**0.5714**	**0.0286**	**0.4426**	0.4571	0.0888	0.6415
**D1 Shared Pathway Genes** ^c^												
IL2RA	0.4021	0.1377	0.7378	0.2393	0.3892	0.8163	0.2107	0.4498	0.8367	0.3000	0.2767	0.7729
**CSF2RB**	0.3181	0.2461	0.7714	0.2929	0.2888	0.7729	0.3893	0.1525	0.7379	**0.5929**	**0.0222**	**0.3933**
FCGR1A	0.4629	0.0838	0.6414	0.3179	0.2479	0.7714	0.3821	0.1607	0.7379	0.4893	0.0666	0.5629
DUSP4	0.0626	0.8249	0.9483	0.2143	0.4421	0.8367	0.4250	0.1159	0.6857	0.1821	0.5150	0.8529
HLA-DQB1	-0.2270	0.4130	0.8189	-0.1929	0.4901	0.8512	-0.1464	0.6023	0.8743	-0.1786	0.5235	0.8529
HLA-DRB1	-0.4147	0.1249	0.7038	-0.2821	0.3074	0.7861	-0.3250	0.2370	0.7714	-0.0036	0.9948	0.9969
**D1 Cytokine Genes** ^d^												
IFNA2	0.3682	0.1763	0.7379	0.1929	0.4901	0.8512	0.5179	0.0506	0.4951	0.3250	0.2370	0.7714
***IL6***	0.2967	0.2807	0.7729	0.2500	0.3678	0.8163	**0.5536**	**0.0349**	**0.4578**	***0.6857***	***0.0061***	***0.3166***
IL18	-0.1948	0.4837	0.8512	-0.2857	0.3011	0.7861	0.5107	0.0543	0.5031	0.1964	0.4819	0.8512
IFNG	0.1537	0.5817	0.8658	-0.2429	0.3820	0.8163	0.4679	0.0808	0.6399	0.2107	0.4498	0.8367
**MCP1**	**0.5326**	**0.0431**	**0.4716**	0.3750	0.1692	0.7379	0.5179	0.0506	0.4951	**0.6393**	**0.0122**	**0.3201**
**D1 Cytokine Proteins**												
IFN-α	-0.1716	0.5380	0.8529	-0.0929	0.7435	0.9302	-0.2750	0.3203	0.7861	0.1464	0.6023	0.8743
IL-6	0.3399	0.2132	0.7714	-0.5076	0.0554	0.5031	-0.4486	0.0948	0.6415	-0.0286	0.9206	0.9871
IL-18	0.1287	0.6455	0.8959	0.0571	0.8425	0.9483	-0.1000	0.7241	0.9287	0.4071	0.1334	0.7295
IFN-γ	0.2952	0.2820	0.7729	-0.2324	0.4016	0.8166	-0.0840	0.7539	0.9302	0.2717	0.3246	0.7861
MCP-1	0.5094	0.0545	0.5031	-0.2286	0.4114	0.8189	0.0571	0.8425	0.9483	0.3357	0.2212	0.7714
IL-1RA	-0.0197	0.9465	0.9907	0.0786	0.7827	0.9414	0.2250	0.4189	0.8212	0.4429	0.1002	0.6657
**D3 DEG**												
CLU	0.1342	0.6452	0.8881	0.4374	0.1198	0.6852	0.0044	0.9911	0.9966	0.1516	0.6051	0.8703
GP1BB	0.0528	0.8587	0.9582	0.4549	0.1044	0.6588	-0.0902	0.7582	0.9305	0.1385	0.6375	0.8872
BCL2L1	0.3410	0.2313	0.7550	0.5209	0.0591	0.8463	0.1782	0.5391	0.8463	0.4769	0.0872	0.6249
IL1RL1	0.2464	0.3929	0.7988	-0.1956	0.5022	0.8424	0.2926	0.3074	0.7734	-0.2440	0.3998	0.7988
ILR2	-0.1276	0.6622	0.8979	-0.1604	0.5838	0.8616	0.0946	0.7467	0.9305	-0.2440	0.3998	0.7988
**D3 Shared Pathway Genes**												
HLA-C	-0.0990	0.7352	0.9305	0.0637	0.8319	0.9532	-0.2332	0.4194	0.8037	0.0593	0.8438	0.9532
FCGR1A	0.3850	0.1736	0.7180	0.0374	0.9035	0.9842	0.4158	0.1396	0.7180	-0.0330	0.9155	0.9911
**D3 Cytokine Genes**												
IFNA2	0.1210	0.6784	0.9030	-0.2747	0.3411	0.7803	0.4422	0.1143	0.6852	0.0242	0.9396	0.9911
IL6	0.3256	0.2543	0.7550	-0.1077	0.7126	0.9288	0.2794	0.3305	0.7741	0.1780	0.5423	0.8463
IL18	0.1716	0.5549	0.8498	-0.3890	0.1703	0.7180	0.1496	0.6076	0.8703	-0.1956	0.5022	0.8424
IFNG	0.0616	0.8349	0.9532	-0.3363	0.2399	0.7550	-0.0572	0.8468	0.9536	-0.1780	0.5423	0.8463
**MCP1**	**0.6161**	**0.0213**	**0.3870**	-0.0154	0.9638	0.9911	0.2444	0.3969	0.7988	0.0418	0.8915	0.9769
IL1RN	0.3476	0.2222	0.7550	0.2528	0.3825	0.7988	-0.0946	0.7466	0.9305	-0.0198	0.9517	0.9911
**D3 Cytokine Proteins**												
IFN-α	-0.2672	0.3737	0.7988	0.0908	0.7678	0.9332	-0.3444	0.2477	0.7550	0.1513	0.6196	0.8824
IL-6	-0.1706	0.5833	0.8616	-0.5154	0.0769	0.6185	0.0088	0.9872	0.9953	-0.4193	0.1666	0.7180
IL-18	0.1828	0.5262	0.8463	0.4004	0.1561	0.7180	-0.0903	0.7579	0.9305	0.3476	0.2221	0.7550
IFN-γ	0.2500	0.3846	0.7988	0.1503	0.6059	0.8703	0.1018	0.7291	0.9288	0.1635	0.5740	0.8616
MCP-1	0.0792	0.7878	0.9412	-0.0637	0.8319	0.9532	-0.1254	0.6673	0.8979	0.0286	0.9276	0.9911
IL-1RA	0.3195	0.2622	0.7550	0.4577	0.1022	0.6565	0.2350	0.4139	0.8014	0.4858	0.0811	0.6185

a bold font corresponds to p < 0.05.

b bold and italic font corresponds to p < 0.01.

c Genes shared between the top five scoring pathways on D1 also included IL1RN, SOCS3, IFITM1, IRF7, NFKBIA, and KIT, genes that are included in the DEG.

d The gene encoding IL-1RA is ILRN that is included in the DEG.

**Figure 8:**
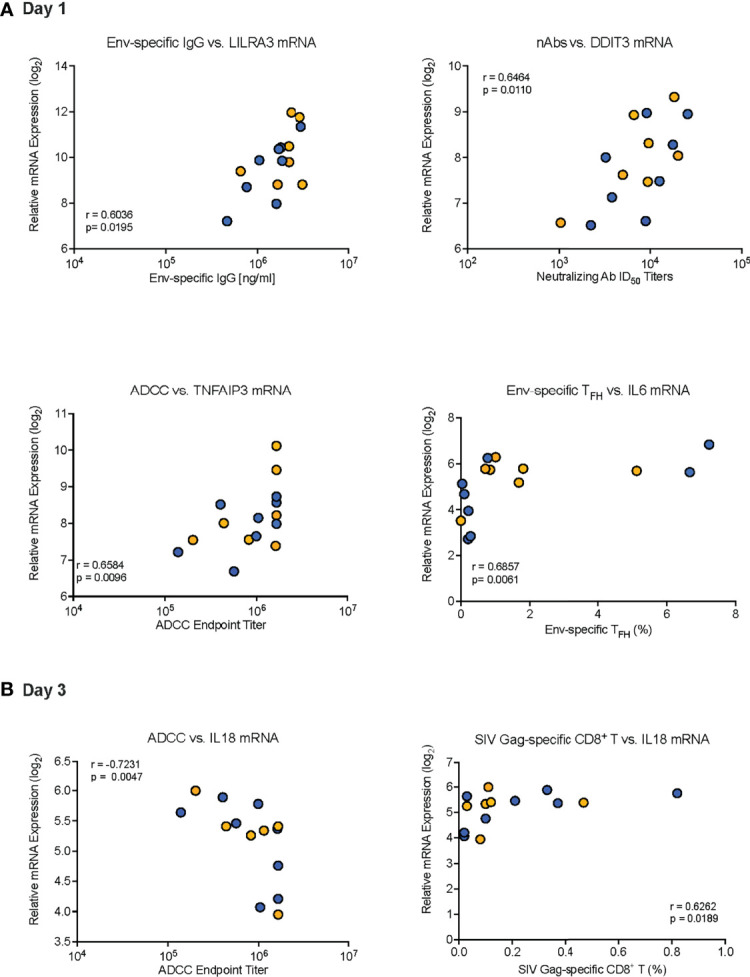
Examples of correlations between day 1 or day 3 innate immune responses and adaptive immune responses. Representative examples of correlations between innate immune responses on Day 1 **(A)** or Day 3 **(B)** with distinct adaptive immune responses are presented. **(A)** Correlation between (i) Env-specific IgG at week 14 and D1 LILRA3 mRNA expression, (ii) neutralizing ID50 titers at week 34 and D1 DDIT3 mRNA expression, (iii) endpoint ADCC titers at week 34 and D1 TNFAIP3 mRNA, and (iv) week 34 Env-specific T_FH_ frequencies and D1 IL6 mRNA expression. **(B)** Correlation between (i) week 34 ADCC endpoint titers and D3 IL18 mRNA levels and (ii) week 34 SIV Gag-specific CD8^+^ T cell frequencies and D3 IL18 mRNA levels. Each symbol represents an individual animal of Group 1 (orange circles) or Group 2 (blue circles). The “r” and “p” values were determined by Spearman rank analysis and represent the correlation coefficient and the unadjusted p values, respectively.

We further evaluated the potential impact of the innate response to the vaccine prime on HIV Env-and SIV Gag-specific CD8^+^ T cell responses in peripheral blood at week 34. SIV Gag-specific CD8^+^ T cell responses were included because both vaccine regimens involved an adenoviral vector prime with ChAdOx1.tSIVconsv239 that was followed by two booster immunizations with MVA.tSIVconsv239 (15, 16). Peripheral blood SIV-Gag-specific CD8^+^ T cell responses (see **Supplemental Figure S3**) at week 34 were negatively correlated to plasma IFN-α concentrations on D1, but positively correlated with mRNA levels of IFNA2 and IL18 on D3 (**Table 6**). Correlations were not observed with peripheral blood HIV Env-specific CD8^+^ T cells (**Table 6**). Representative examples of correlations between vaccine prime-induced innate immune responses and adaptive immune responses are provided in **Figure 8**.

**Table 6:** Correlation of early gene expression with antigen-specific CD8^+^ T cell responses.

Parameter	SIV Gag-specific CD8^+^ T			HIV Env-specific CD8^+^ T		
	Spearman Correlation^a^					
	r	p	q	r	p	q
**D1 DEG**						
SERPING1	0.2272	0.4123	0.8014	-0.1841	0.5085	0.8460
C3AR1	-0.0966	0.7308	0.9288	-0.3378	0.2170	0.7550
IL1RN	0.0322	0.9106	0.9889	-0.2985	0.2779	0.7550
TNFAIP6	-0.0125	0.9667	0.9911	-0.1341	0.6319	0.8861
PLAUR	0.0575	0.8397	0.9532	0.0518	0.8551	0.9571
FPR2	0.1306	0.6406	0.8881	0.0572	0.8396	0.9532
SOCS3	0.2666	0.3342	0.7741	-0.3146	0.2516	0.7550
OAS1	0.0286	0.9208	0.9911	-0.1528	0.5729	0.8616
TNFSF10	0.1073	0.7019	0.9241	-0.2931	0.2867	0.7550
CCR1	-0.2111	0.4471	0.8274	-0.3682	0.1764	0.7181
LILRA3	0.0859	0.7601	0.9305	-0.0089	0.9771	0.9953
IFITM1	-0.0054	0.9872	0.9953	-0.3450	0.2070	0.7550
ARG2	0.1699	0.5419	0.8463	0.0107	0.9717	0.9934
CD82	0.2039	0.4629	0.8346	-0.0232	0.9363	0.9911
IRF7	-0.0608	0.8296	0.9532	-0.2055	0.4595	0.8341
IFI35	0.1127	0.6875	0.9117	-0.1323	0.6362	0.8872
NFKBIA	0.0376	0.8952	0.9781	-0.0822	0.7700	0.9332
CSF3R	-0.0590	0.8346	0.9532	-0.1787	0.5209	0.8463
DDIT3	0.0072	0.9821	0.9953	-0.1430	0.6088	0.8703
GP1BB	0.0877	0.7551	0.9305	0.2752	0.3181	0.7734
CLU	0.2326	0.4012	06852	0.3592	0.1878	0.7393
TNFAIP3	0.0250	0.9309	0.9911	-0.3825	0.1592	0.7180
IL1RL1	0.0125	0.9667	0.9911	-0.1984	0.4756	0.8413
HLA-DMA	-0.3757	0.1673	0.1780	0.0214	0.9410	0.9911
KIT	0.0734	0.7946	0.9412	0.2752	0.3181	0.7734
**D1 Shared Pathway Genes** ^c^						
IL2RA	-0.0447	0.8749	0.9675	0.1233	0.6598	0.8979
CSF2RB	-0.0143	0.9616	0.9911	-0.1930	0.4875	0.8413
FCGR1A	-0.0054	0.9872	0.9953	-0.1984	0.4756	0.8413
DUSP4	-0.1002	0.7212	0.9288	-0.1519	0.5865	0.8621
HLA-DQB1	-0.1342	0.6313	0.8861	0.2359	0.3942	0.7988
HLA-DRB1	-0.2701	0.3275	0.7734	-0.2359	0.3942	0.7988
**D1 Cytokine Genes** ^d^						
IFNA2	0.1664	0.5506	0.8477	0.3146	0.2516	0.7550
IL6	0.0572	0.8397	0.9532	0.1215	0.6641	0.8979
IL18	0.1252	0.6546	0.8973	-0.1734	0.5340	0.8463
IFNG	0.2630	0.3409	0.7803	0.2073	0.4552	0.8332
MCP1	-0.0805	0.7748	0.9359	-0.1912	0.4919	0.8413
**D1 Cytokine Proteins**						
**IFN-α**	**-0.6440**	**0.0113**	**0.3149**	0.2431	0.3797	0.7988
IL-6	0.0161	0.9553	0.9911	0.1682	0.5434	0.8463
IL-18	-0.3846	0.1567	0.7180	0.5004	0.0593	0.5055
IFN-γ	-0.0877	0.7539	0.9305	0.0734	0.7943	0.9412
MCP-1	0.1360	0.6268	0.8861	-0.0590	0.8350	0.9532
IL-1RA	-0.0608	0.8296	0.9532	0.3199	0.2435	0.7550
**D3 DEG**						
CLU	-0.0022	0.9969	0.9969	0.1696	0.5596	0.8533
GP1BB	-0.1411	0.6281	0.8861	0.1960	0.4989	0.8424
BCL2L1	0.1676	0.5641	0.8567	0.1234	0.6727	0.8997
IL1RL1	0.3462	0.2240	0.7550	0.1256	0.6672	0.8979
ILR2	-0.1147	0.6948	0.9180	-0.0154	0.9607	0.9911
**D3 Shared Pathway Genes**						
HLA-C	-0.2272	0.4316	0.8185	0.0771	0.7933	0.9412
FCGR1A	0.3664	0.2170	0.7550	-0.3106	0.2778	0.7550
**D3 Cytokine Genes**						
**IFNA2**	**0.6615**	**0.0119**	**0.3149**	0.2643	0.3583	0.7988
IL6	0.4851	0.0806	0.6185	0.0793	0.7873	0.9412
**IL18**	**0.6262**	**0.0189**	**0.3641**	0.3811	0.1785	0.7180
IFNG	0.1610	0.5798	0.8616	0.2137	0.4603	0.8341
MCP1	0.2315	0.4226	0.8055	0.0551	0.8525	0.9571
IL1RN	0.0220	0.9424	0.9911	0.0440	0.8822	0.9726
**D3 Cytokine Proteins**						
IFN-α	-0.2666	0.3742	0.7988	0.3200	0.2833	07550
IL-6	0.2851	0.3718	0.7988	0.1051	0.7308	0.9288
IL-18	-0.1004	0.7302	0.9288	0.2183	0.4494	0.8274
IFN-γ	0.2661	0.3538	0.7988	0.1008	0.7279	0.9288
MCP-1	0.1874	0.5180	0.8463	0.3040	0.2885	0.7550
IL-1RA	0.0494	0.8672	0.9618	-0.0141	0.9643	0.9911

a bold font corresponds to p < 0.05.

b bold and italic font corresponds to p < 0.01.

c Genes shared between the top five scoring pathways on D1 also included IL1RN, SOCS3, IFITM1, IRF7, NFKBIA, and KIT, genes that are included in the DEG.

d The gene encoding IL-1RA is ILRN that is included in the DEG.

Although none of the correlations remained statistically significant after adjusting for multiple comparison testing at the 0.05 significance level, the fact that correlations of early DEG mRNA with Env-specific ADCC responses were almost exclusively observed at week 34, suggested that these associations were non-random. Additionally, the distribution of unadjusted p-values in each hypothesis group (**Supplemental Figure S6**) is favored more heavily in lower p-values. As the tests for moderate correlations were underpowered given the current sample size, this shape suggested qualitatively that there may be potential true correlation estimates that were not detectable as statistically significant in this study due to lack of power after FDR correction.

**Figure 9:**
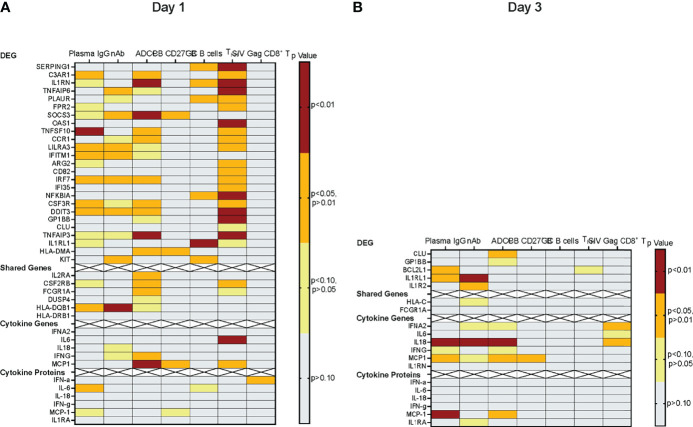
Correlations of early vaccine prime-induced and vaccine-induced adaptive immune responses. Correlations for D1 or D3 are illustrated in Panels **(A)** or **(B)**, respectively. The left y-axis lists the various genes or proteins included in the correlation analyses: (i) DEG, (ii) shared pathway genes, (iii) genes of elevated plasma cytokines, or (iv) plasma cytokines. The strength of the correlation between gene expression and a specific immune parameter is indicated by a heatmap using the relevant Spearman rank correlation unadjusted p values (see color legend). Each column represents a specific immune response, including - from left to right– Env-specific plasma IgG, tier 1 MW965-specific neutralizing antibody ID_50_ titers, ADCC endpoint titers, peripheral blood memory B cells at week 34, germinal center B cells in axillary lymph nodes at week 34, Env-specific follicular T helper cells in axillary lymph nodes at week 34, and SIV Gag-specific peripheral blood CD8^+^ T cell responses at week 34.

In summary, we discovered several correlations between early vaccine prime-induced immune responses and vaccine-induced adaptive immune parameters (**Figure 9**). The induction of several D1 DEG appeared to promote Env-specific antibody responses, whereas elevated plasma cytokines on D1 inversely affected antibody responses. The most pronounced effect of the D1 innate responses was on Env-specific T_FH_ (**Figure 9**). In agreement with the transient upregulation of genes, by D3 positive correlations between gene expression and vaccine-induced antibody responses were no longer detectable. The mRNA levels of most cytokine-encoding genes were inversely correlated with Env-specific plasma IgG, neutralizing antibodies, and ADCC responses (**Figure 9**). Furthermore, among the genes with altered expression on D1, a subset of eight genes (IL1RN, CCR1, TNFAIP3, HLA-DMA, IL2RA, CSF2RB, FCGR1A, and MCP1; **Table 4**) only correlated with ADCC function, but not with Env-specific IgG or neutralizing antibodies.

### Discussion

The results of the current study demonstrate that the vaccine prime with an HIV Env protein mixed with 3M-052-SE induced a rapid, but transient, innate immune response characterized by an increase in inflammatory cytokines and elevated mRNA levels of genes associated with chemotaxis, type I interferon responses, and the sensing and priming of adaptive immune responses. Our results also suggest that the inclusion of the MVA-HIV vaccine in addition to the HIV Env protein vaccine modified this inflammatory response. Nonetheless, the mRNA levels of differentially expressed genes on day 1 in animals of both groups correlated with the magnitude and function of vaccine-induced adaptive immune responses assessed between weeks 14 to 34 post prime. The latter finding is consistent with other studies documenting a link between early innate immune responses and vaccine-induced immunogenicity at later timepoints (8, 10, 13, 14, 31).

Relevant to the current study, a recent analysis of samples from the RV144 HIV vaccine trial in human adults found that several genes were upregulated on day 1 after the vaccine prime, and then, analogous to our results, rapidly returned to baseline (pre-vaccine) levels (14). In addition, the authors noted an increase in several plasma cytokines on day 1. Among those cytokines were IL-6, MCP-2, and IFN-γ, cytokines that were also found at elevated plasma levels in our study. In RV144 participants, increases in cytokines at day 1 were positively correlated with the vaccine-induced Env-specific plasma antibodies at 6.5 months, whereas the early gene signature was not correlated with plasma Env-specific IgG responses (14). The early gene signature was, however, positively correlated with ADCC and antibody-dependent phagocytosis function at 6.5 months (14). In our study, with the exception of IFN-α that was inversely correlated with SIV Gag-specific CD8^+^ T cell responses, we did not find a correlation between day 1 elevated plasma cytokines and vaccine-induced adaptive immune responses between weeks 14 to 34. However, on D3 IFN-α and IL-18 plasma concentrations were positively correlated with SIV Gag-specific CD8^+^ T cell responses, while MCP-1 was inversely correlated to plasma IgG and ADCC function.

Similar to the findings in the RV144 analysis, several of the DEG identified in the current study were positively correlated with Env-specific antibody responses. It was notable that the correlation of early genes with the magnitude of Env-specific ADCC responses was primarily found at week 34, a result consistent with maturation of functional antibody responses over time. This question should be addressed in future studies to determine at what timepoint correlations between early vaccine-prime-induced responses and specific functional adaptive immune parameters should be assessed to predict vaccine immunogenicity and potential efficacy. We identified several genes that were only correlated with ADCC, but not with plasma IgG and neutralizing antibody responses. A study analyzing the transcriptome of adult rhesus macaques vaccinated with a mosaic adenovirus 26-based SIV vaccine that provided partial efficacy against infection with SIV and/or SHIV challenge identified a specific B cell signature that was associated with immune correlates of protection (32). Importantly, this molecular signature could be validated in human adult participants of the RV144 trial that were protected against HIV acquisition (32). One of the genes, TNFSF13, correlated specifically with ADCC and ADCP responses in vaccinees. The D1 mRNA levels of the related genes TNFSF10, TNFIAP3, and TNFIAP6 were also correlated with ADCC responses in the current study. Conversely, we need to determine whether functionally distinct adaptive immune responses can be foretold by specific innate immune or molecular signatures. This question is important for HIV vaccine design to modulate innate immune responses in a targeted fashion to optimize the induction of broadly neutralizing antibodies, antibodies with Fc-mediated effector function, and/or antiviral T cell responses.

Earlier studies have demonstrated that distinct vaccine strategies (live attenuated versus inactivated viral vaccines versus polysaccharide vaccines) differ in the early immune response (9). A comparative study examining the early peripheral blood transcriptome in response to five different vaccines found that despite vaccine-specific gene signatures, similar innate immune response pathways, such as complement activation, inflammation, and antigen-sensing and presentation, were targeted (10). The DEG identified in the current study support these earlier findings. Adjuvants are important means in modulating the early innate response and enhancing specific adaptive immune responses (7, 33). In fact, we and others have previously reported how different adjuvants can alter the magnitude and quality of HIV Env-specific antibody responses (34–36). In the current study, several of the DEG (e.g., the type I interferon inducible genes OAS1, IRF7, IFITM1, SOCS3, IFI35) likely reflected the host response to the TLR7/8-agonist-based adjuvant 3M-052-SE (37–39). This conclusion was supported when a network and enrichment analysis that included all D1 genes that were positively correlated with antibody responses analysis identified the TLR7/8 cascade as one of the important pathways (FDR p=2.01E-08) (**Supplemental Figure S7**). When we limited the analysis to genes that were only correlated with ADCC function, the Toll-like receptor signaling pathway (FDR p=6.36E-06), the BCR signaling pathway (FDR p=0.0002), and the NK cell-mediated cytotoxicity pathway (FDR p=0.0028) were also identified in the enrichment analysis (**Supplemental Table S5**). It should be noted though that enrichment analysis of the data in our study here is biased as we performed a targeted transcriptome analysis and therefore only a limited number of genes could be identified.

The type I interferon response could have also been induced by the administration of the viral ChAd vector (40–43). The coadministration of MVA in Group 2 appeared to modify the innate response observed in Group 1. The current study did not include an adjuvant only group or groups being only immunized with the ChAd vector or MVA. Therefore, we were not able to study the innate response to the individual vaccine components (adjuvant, ChAdV, or MVA). As the individual components of the vaccine regimen were administered at different sites, the potential interference in innate immune responses must have been due to a rapid systemic effect. Interestingly, it was reported previously that the simultaneous administration of HAdV5 and MVA vectors resulted in vaccine interference, evident in suppressed CD8^+^ T cell responses (44). It is also well established that MVA encodes several immune evasion genes that could have suppressed the innate response, especially the type I interferon response, induced by the 3M-052-SE adjuvanted HIV Env protein (45, 46). In fact, novel MVA vaccine vectors are being developed to improve the immunogenicity of MVA (47, 48). However, it is also well documented that MVA can induce potent innate responses (49–51). In fact, animals in Group 2 had increased plasma concentrations of several inflammatory cytokines on day 1. It is plausible that the induction of genes in response to the vaccine prime peaked prior to our first sampling timepoint at 24 hours in Group 2. Alternatively, the kinetics of mRNA induction in Group 2 could have been delayed and occurred between day 1 and day 3. In fact, in Group 2 animals some cytokines were still elevated on day 3 compared to day 0. Furthermore, specific genes and proteins are induced by distinct cell types and, thus, changes in relative frequencies of peripheral blood cell populations on days 1 and 3 compared to day 0 could have altered results obtained in the whole blood gene expression analysis. Our findings emphasize the need to assess the impact of individual vaccine components on vaccine immunogenicity at a more granular level, including in individual cell populations and over a more frequent sampling interval.

Both the induction of type I interferons and the activation of Myd88 by TLR-based adjuvants have been demonstrated to enhance the inflammatory response through NF-kB activation (7, 52–54). TLR signaling has also been linked to germinal center formation, isotype switching, and antibody maturation (55, 56). While TLR agonists can directly activate B cells, they also indirectly enhance antibody responses through the activation of T_FH_ [reviewed in (23)]. In fact, the D1 expression of several DEG in the current study were correlated with Env-specific lymph node T_FH_ frequencies at week 34 (**Table 6**). Although we found few correlations between DEG and memory or GC B cells at week 34 (**Table 6**), it should be noted that we measured total memory and GC B cells and not Env-specific B cells.

Due to the limited sample size, we could not validate our findings in a different study setting or with a different data set. Furthermore, because we did not challenge the animals, we do not know whether and how the correlations between early gene induction and vaccine-induced adaptive immune responses inform predictions about vaccine efficacy. However, the conclusion that the observed correlations between early innate immune signatures and later vaccine-induced adaptive immune responses are biologically relevant is supported by data demonstrating the role of complement (57), type I interferons (58, 59), IL-6 (60–62), and TLR signaling (55, 56) in B cell activation and maturation. The result of the network and enrichment analyses with genes that were associated with one or more adaptive immune parameters that identified predicted partners involved in the BCR signaling pathway further substantiated this conclusion (**Supplementary Figure X**). Overall, the results support the idea that through modulation of innate immune responses by targeted modifications in the vaccine prime, we can direct specific HIV-specific antibody and T cell responses to optimize vaccine-induced immunity. The latter might be especially important for pediatric vaccines due to the dynamic nature of the immune systems during neonatal and infant development.

## Data Availability Statement

The original contributions presented in the study are included in the article/supplementary files, further inquiries can be directed to the corresponding author.

## Ethics Statement

The animal study was reviewed and approved by UC Davis Institutional Animal Care and Use Committee.

## Author Contributions

KDP and SRP conceived and planned the study. KKVV and KDP wrote the manuscript. KKVV, ADC, and JP performed the experiments and analyzed the data. KKAVR oversaw the animal studies. KAC and MGH conducted the statistical analysis. MT and CF provided the 3M-052-SE adjuvant, and TH the ChAdOx1t.SIVcons239 and MVA.tSIVconsv239 vaccines. All authors critically reviewed and edited the manuscript. All authors contributed to the article and approved the submitted version.

## Funding

The work was supported by National Institutes of Health grants 1R01 DE028146 (KDP), P01 AI117915 (SRP, KDP), T32 5108303 (ADC), the Office of Research Infrastructure Programs/OD P510D011107 (CNPRC), and the Center for AIDS Research award P30AI050410 (UNC). The UNC Flow Cytometry Core Facility is supported in part by P30 CA016086 Cancer Center Core Support Grant to the UNC Lineberger Comprehensive Cancer Center. Research reported in this publication was supported by the Center for AIDS Research award number 5P30AI050410.

## Author Disclaimer

The content is solely the responsibility of the authors and does not necessarily represent the official views of the National Institutes of Health.

## Conflict of Interest

Author MT was employed by the company 3M Corporate Research Materials Laboratory.

The remaining authors declare that the research was conducted in the absence of any commercial or financial relationships that could be construed as a potential conflict of interest.

## Publisher’s Note

All claims expressed in this article are solely those of the authors and do not necessarily represent those of their affiliated organizations, or those of the publisher, the editors and the reviewers. Any product that may be evaluated in this article, or claim that may be made by its manufacturer, is not guaranteed or endorsed by the publisher.

## Supplementary Material

Supplementary Figure 1 Assessment of Memory and GC B Cell Frequencies by Flow Cytometry. Tissue samples were processed into single cell suspensions and stained with surface markers prior to fixation and analysis *via* flow cytometry. Only events in the R1 lymphocyte gate were recorded (Top left). Single live lymphocytes that were negative for lineage markers CD3, CD14 and CD16 (top right) were assessed for CD20 and CD27 expression. CD20+CD27- B (bottom right) and CD20+CD27+ memory B (bottom left) cells were analyzed for expression of CXCR5 (CD185).

Supplementary Figure 2 Gating strategy for Env-specific follicular T helper cells. Samples were cultured for 18-24 hours in the presence of vehicle DMSO (negative control), Staphylococcal enterotoxin B (SEB) (positive control), or HIV-1 Clade C Consensus Peptide Pool and analyzed by flow cytometry for surface expression of activation induced T_FH_ markers. Top row: Viable CD3^+^ lymphocyte events were assessed for the presence of CD4 (thick black gate). Middle row: CD4^+^ events were further classified as follicular T cells based on the presence of CD185 (CXCR5) and CD279 (PD-1) signal (blue gate). Activation Induced Marker (AIM) T follicular helper cells were defined as CD134^+^CD137^+^ events (magenta gate). Env-specific AIM T_FH_ were calculated by normalizing the frequency against the DMSO control samples.

Supplementary Figure 3 Flow cytometric identification of SIV Gag-specific CD8^+^ T cells. Samples were stimulated with media only (negative control), PMA/Ionomycin (positive control) or overlapping peptides of SIV p27 Gag for 6 hours in the presence of Brefeldin-A for the final 5 hours. Samples were stained with surface markers and subsequently fixed, permeabilized, and subjected to intracellular staining. Top row: viable events from the lymphocyte gate were assessed for CD3 and CD8. Gating for TNF-α, IFN-γ, IL-2 or IL-17 is shown for each experimental treatment. SIV Gag-specific responses were reported as the frequency of single-stained (Boolean logic) cytokine-positive events normalized to the frequency of the corresponding ‘media only’ control sample.

Supplementary Figure 4 Gene expression analysis of Group 1 and Group 2 prior and post vaccination. *Panel A:* Principal component plot of PC1 and PC2 mRNA data of D0 samples from Group 1 (red circles) and Group 2 (blue circles). Image was generated using Clustviz.

Supplementary Figure 5 Interaction network of differentially expressed genes. The network was created in Cytoscape as degree-directed layout using the Human String Protein Database. Interactions between the individual proteins are indicated by connecting lines. Note that for the D1 DEG LILAR3, ARG2, CD82, DDIT3, GP1BB, and HLA-DMA no interactions were identified.

Supplementary Figure 6 Distribution of unadjusted p-values from two-sided tests for Spearman correlations. **(A, B)** show the distribution of unadjusted p-values from two-sided tests for Spearman correlations (H_a_: rho ≠ 0) between differentially regulated transcripts and **(A)** humoral (Env-specific plasma IgG, ADCC, and neutralizing antibody responses; total tests: n=661) or **(B)** cellular (memory B and germinal center B cells, T_FH_ cells, and HIV Env- and SIV Gag-specific CD8^+^ T-cells; total tests: n=366) immune responses. Unadjusted p values in 0.05 increments are listed on the x-axis. The y-axis lists the percentage of unadjusted p values falling into the range of each increment. The number on top of each bar represents the absolute number of tests within each p value range. The dashed line indicates the expected percentage if correlations were randomly distributed.

Supplementary Figure 7 Potential interactions between day 1 induced genes and specific signaling pathways. Genes that were increased on D1 and correlated to vaccine-induced antibody responses were entered into NetworkAnalyst to assemble a network based on the String v11 Human Interactome. Major hubs (nodes) are indicated by red and orange circles. Predicted interactions between the genes are indicated by edges (black lines), with dark blue circles symbolizing predicted interaction partners. In Panels A and B genes that are part of the KEGG B cell receptor or the TLR7/8 signaling pathway, respectively, are represented by light blue circles.
