# Supplementary material for: Early Post-Vaccination Gene Signatures Correlate With the Magnitude and Function of Vaccine-Induced HIV Envelope-Specific Plasma Antibodies in Infant Rhesus Macaques
Source: Front Immunol. 2022 Apr 27;13:840976. doi: 10.3389/fimmu.2022.840976 (PMC9094446; doi:10.3389/fimmu.2022.840976)
Supplement: Supplementary Figure 1 — Assessment of Memory and GC B Cell Frequencies by Flow Cytometry. Tissue samples were processed into single cell suspensions and stained with surface markers prior to fixation and analysis via flow cytometry. Only events in the R1 lymphocyte gate were recorded (Top left). Single live lymphocytes that were negative for lineage markers CD3, CD14 and CD16 (top right) were assessed for CD20 and CD27 expression. CD20+CD27- B (bottom right) and CD20+CD27+ memory B (bottom left) cells were analyzed for expression of CXCR5 (CD185). [file DataSheet_1.pdf]

Supplemental Table 1: Differentially expressed genes and their function

Gene	Name	Pathway[a]	Function
**Day 1 Upregulation**			
C3AR1	C3a	complement	anaphylatoxin chemotactic receptor
SERPING1	serine protease inhibitor	complement	plasma protease C1 inhibitor
OAS1	2'-5'-oligoadenylate synthase 1	interferon signaling	innate antiviral responses
SOCS3	suppressor of cytokine signaling 3	interferon and interleukin signaling, adaptive immunity	
IFI35	interferon-inducible protein 35	interferon signaling	NF-kB activation
IFITM1	interferon-induced transmembrane protein 1	interferon signaling, adaptive immunity	antiviral function
IRF7	interferon-responsive factor 7	interferon signaling, innate immunity, TLR signaling	
IL1RN	interleukin 1 receptor antagonist	interleukin signaling	inhibition of IL-1
NFKBIA	NF-kB inhibitor alpha	interleukin signaling, innate immunity, adaptive immunity, FcR signaling, NF-kB signaling, TLR TLR signaling	
LILRA3	leukocyte immunoglobulin-like receptor A3	adaptive immunity	regulation of immune activation
ARG2	arginase 2	metabolism	
CCR1	C-C chemokine receptor 1		receptor for MIP-1α, MIP-1β, MCP-1, RANTES
TNFSF10	TNF superfamily member 10		apoptosis
TNFAIP3	TNF-a-inducible protein 3		cell-cell interactions, inflammation
TNFAIP6	TNF-a-inducible protein 6		cell-cell interactions, inflammation
GP1BB	platelet glycoprotein 1b beta chain		platelet surface protein, adhesion
CSF3R	colony-stimulating factor 3 receptor		important in innate immunity and neutrophil function
FPR2	N-formyl peptide receptor 2		low affinity receptor for neutrophil chemotactic factors
CD82	CD82 antigen		T cell costimulation, tetraspanin
CLU	clusterin		chaperone for protein folding, apoptosis
DDIT3	DNA damage-inducible transcript 3		CCAAT/ enhancer- binding protein C/EBP) family of transcription factors
**Day 1 Down-Regulation**			
KIT	tyrosine receptor kinase III family receptor for mast/stem cell growth factor	adaptive immunity, Fc receptor signaling, interleukin signaling	regulation of proliferation and cell survival, innate immunity, MAPK, VEGF signaling
IL1RL1	interleukin 1 receptor like 1	interleukin signaling	inflammation
HLA-DMA	HLA class II alpha		antigen presentation

Table 2: Differentially expressed genes and their function

Gene	Name	Pathway[a]	Function
**Day 3 Upregulation**			
GP1BB	glycoprotein 1b platelet subunit beta		formation of platelet plugs
CLU	clusterin		chaperone for protein folding, apoptosis
**Day 3 Downregulation**			
IL-1RL1	interleukin 1 receptor like 1	interleukin signaling	inflammation
IL1R2	interleukin 1 receptor 2	interleukin signaling	inflammation
BCL2L1	BCL-2 -like protein		apoptosis

[a] Pathway(s) of the Nanostring NHP Immunology Panel that contain(s) the specific gene. If no pathway is listed, the gene remained unassigned in the Nanostring analysis.

Supplemental Table S2: Differentially expressed genes on Day 1 and Day 3

Gene	Group 1 mRNA Expression				Group 2 mRNA Expression			
	D1/D0[a]		D3/D0[b]		D1/D0		D3/D0	
	log_2	p[c]	log_2	p	log_2	p	log_2	p
**Day 1 Up-Regulation**								
SERPING1	**3.29**	**0.0291**	-0.19	1.0000	1.71	1.0000	1.11	1.0000
C3AR1	**3.12**	**0.0211**	-0.53	1.0000	1.24	1.0000	-0.17	1.0000
IL1RN	**2.78**	**0.0358**	-2.93	0.1640	1.68	1.0000	-0.38	1.0000
TNFAIP6	**2.67**	**0.0135**	-1.03	1.0000	1.63	0.9190	0.39	1.0000
PLAUR	**2.22**	**0.0629**	-2.00	0.4830	0.33	1.0000	-1.33	1.0000
FPR2	**2.15**	**0.0427**	-1.40	1.0000	1.10	1.0000	-0.29	1.0000
SOCS3	**2.11**	**0.0550**	-0.99	1.0000	1.02	1.0000	0.28	1.0000
OAS1	**2.06**	**0.0550**	-1.17	1.0000	1.44	1.0000	0.79	1.0000
TNFSF10	**1.98**	**0.0135**	-0.07	1.0000	0.84	1.0000	0.37	1.0000
CCR1	**1.94**	**0.0427**	-1.06	1.0000	1.01	1.0000	0.21	1.0000
LILAR3	**1.83**	**0.0870**	-0.98	1.0000	1.11	1.0000	-0.26	1.0000
IFITM1	**1.79**	**0.0135**	-0.20	1.0000	0.81	1.0000	0.13	1.0000
ARG2	**1.78**	**0.0974**	-0.88	1.0000	1.04	1.0000	-0.22	1.0000
CD82	**1.72**	**0.0334**	-0.12	1.0000	1.03	1.0000	0.03	1.0000
IRF7	**1.64**	**0.0881**	-1.27	0.9150	1.03	1.0000	0.25	1.0000
IFI35	**1.57**	**0.0701**	-0.82	1.0000	0.69	1.0000	0.16	1.0000
NFKBIA	**1.48**	**0.0427**	-0.64	1.0000	0.53	1.0000	-0.14	1.0000
CSF3R	**1.48**	**0.0950**	-0.92	1.0000	0.14	1.0000	-0.28	1.0000
DDIT3	**1.45**	**0.0725**	0.01	1.0000	1.14	1.0000	0.80	1.0000
GP1BB	**1.43**	**0.0427**	1.88	0.0191	0.49	1.0000	1.06	1.0000
CLU	**1.42**	**0.0427**	1.88	0.0191	0.78	1.0000	1.12	0.9010
TNFAIP3	**1.32**	**0.0358**	-0.21	1.0000	0.61	1.0000	0.13	1.0000
**Day 1 Down-Regulation**								
IL1RL1	-1.45	0.3620	-1.77	0.5920	**-2.66**	**0.0250**	-2.36	0.0920
HLA-DMA	**-1.51**	**0.0427**	-0.04	1.0000	-1.06	1.0000	-0.36	1.0000
KIT	**-2.29**	**0.0512**	-0.80	1.0000	**-3.14**	**0.0090**	-1.68	1.0000
**Day 3 Up-Regulation**								
CLU	1.42	0.0427	**1.88**	**0.0191**	0.78	1.0000	1.12	0.9010
GP1BB	1.43	0.0427	**1.88**	**0.0191**	0.49	1.0000	1.06	1.0000
**Day 3 Down-Regulation**								
BCL2L1	0.23	0.0093	-0.01	1.0000	-0.94	1.0000	**-1.62**	**0.0800**
IL1RL1	-1.45	0.3620	-1.77	0.5920	-2.66	0.0090	**-2.36**	**0.0920**
IL1R2	-0.25	1.0000	-1.98	0.4390	-1.40	1.0000	**-2.45**	**0.0800**

[a] D1/D0 = Fold-change in gene expression on day 1 compared to day 0

[b] D3/D0 = Fold-change in gene expression on day 1 compared to day 0

[c] The p value correspond to the adjusted p value determined using the Benjamini-Yekutieli method to correct for multiple comparisons.

Supplemental Table S3: The number of detected, either up- or downregulated, genes in each of the pathway

Pathway	Number of detected genes/ total number of genes
Adaptive immunity	123/144
Apoptosis	35/ 36
Cell cycle	19/ 19
Cellular stress	27/ 31
Complement system	14/ 34
Death receptor signaling	16/ 18
Extracellular organization	27/ 40
Fc receptor signaling	60/ 73
Innate immunity	21/ 21
Interferon signaling	82/ 90
Interleukin signaling	89/123
MAPK signaling	49/ 65
Metabolism	29/ 42
NFκB	35/ 45
TLR signaling	66/ 68
VEGF signaling	42/ 65
Wnt signaling	19/ 20

Supplementary Table S4: Pathway Genes

Pathway	Gene	Group 1		Group 2	
		log_2	BY[a] p Value	log_2	BY[a] p Value
**D1[b] Interferon**	SOCS3	2.11	0.055	1.02	1.000
	OAS1	2.06	0.055	1.44	1.000
	IFITM1	1.79	0.014	0.81	1.000
	OASL	1.75	0.165	0.86	1.000
	OAS3	1.74	0.333	0.48	1.000
	IRF7	1.64	0.088	1.03	1.000
	MX1	1.58	0.149	0.79	1.000
	DDX58	1.58	0.146	0.78	1.000
	IFI35	1.57	0.070	0.69	1.000
	IFIT2	1.56	0.336	0.95	1.000
	GBP1	1.55	0.165	0.58	1.000
	IFIT1	1.52	0.240	0.76	1.000
	IFIT3	1.48	0.191	0.89	1.000
	NFKBIA	1.48	0.043	0.52	1.000
	OAS2	1.41	0.125	0.91	1.000
	FCGR1A	1.37	0.433	1.01	1.000
	MX2	1.37	0.240	0.52	1.000
	TNFAIP3	1.32	0.036	0.61	1.000
	HLA-DQB1	-1.60	0.220	-0.90	1.000
	HLA-DRB1	-1.73	0.156	-1.07	1.000
**D1 Adaptive Immunity**	SOCS3	2.11	0.055	1.02	1.000
	SIGLEC1	2.08	0.165	1.12	1.000
	LILRA3	1.83	0.087	1.11	1.000
	IFITM1	1.79	0.014	0.81	1.000
	CD274	1.76	0.174	0.89	1.000
	NFKBIA	1.48	0.043	0.52	1.000
	FCGR1A	1.37	0.433	1.01	1.000
	PDCD1LG2	1.37	1.000	1.49	1.000
	HLA-DMB	-1.55	0.365	-1.37	1.000
	HLA-DQB1	-1.60	0.220	-0.90	1.000
	HLA-DRB1	-1.73	0.156	-1.07	1.000
	KIT	-2.29	0.051	-3.14	0.009
**D1 Interleukin**	IL1RN	2.78	0.036	1.68	1.000
	IL18RAP	2.16	0.354	1.78	1.000
	SOCS3	2.11	0.055	1.02	1.000
	IL1RAP	1.41	0.172	0.85	1.000
	CSF2RB	1.41	0.125	0.01	1.000
	IL2RA	1.34	0.301	1.09	1.000
	DUSP4	-1.41	0.722	-0.31	1.000
	IL1RL1	-1.45	0.362	-2.66	0.025
	IL23A	-2.18	0.131	-0.74	1.000
	KIT	-2.29	0.051	-3.14	0.009
	IL1R2	-0.25	1.000	-1.40	1.000
	RAPGEF2	-0.13	1.000	-1.38	1.000
**D1 TLR**	IRF7	1.64	0.881	1.03	1.000
	NFKBIA	1.48	0.043	0.52	1.000
	TLR5	1.37	0.153	0.68	1.000
	TLR3	0.72	1.000	1.49	1.000
	DUSP4	-1.41	0.722	-0.31	1.000

**D1 FcG Receptor**	NFKBIA	1.48	0.043	0.52	1.000
	CSF2RB	1.41	0.125	0.01	1.000
	IL2RA	1.34	0.301	1.09	1.000
	DUSP4	-1.41	0.722	-0.31	1.000
	FCGER1A	-1.43	0.416	-1.38	1.000
	KIT	-2.29	0.051	-3.14	0.009
**D3[c] Interferon**	OAS3	-1.34	1.000	0.17	1.000
	FCGR1A	-1.42	1.000	-0.31	1.000
	HLA-C	-1.43	1.000	3.69	1.000
	IFIT3	-1.43	0.813	0.35	1.000
	DDX58	-1.43	0.813	0.25	1.000
	IFIT1	-1.63	0.736	0.26	1.000
	IFIT2	-2.29	0.001	0.13	1.000
**D3 Adaptive Immunity**	FCGR1A	-1.42	1.000	-0.31	1.000
	HLA-C	-1.43	1.000	3.69	1.000
	KIT	-0.80	1.000	-1.68	1.000
**D3 Interleukin**	ITGA2B	1.63	0.189	0.71	1.000
	IL13RA1	-1.39	0.565	-0.93	1.000
	IL1B	-1.50	0.813	-0.46	1.000
	IL1RAP	-1.60	0.439	-0.74	1.000
	IL1RL1	-1.77	0.592	-2.36	0.092
	IL1R2	-1.98	0.439	-2.45	0.080
	IL8	-2.29	0.447	-0.80	1.000
	IL18R1	-2.31	0.189	-1.76	0.901
	IL1RN	-2.93	0.164	-0.38	1.000
	IL18RAP	-3.50	0.189	-0.74	1.000
	KIT	-0.80	1.000	-1.68	1.000
**D3 TLR**	TLR4	-1.49	0.371	-0.66	1.000
	TLR3	-2.03	1.000	0.78	1.000
	TLR7	0.52	1.000	1.46	1.000
**D3 Metabolism**	PTGS1	1.67	0.357	0.95	1.000
	ABCB1	-1.72	1.000	-0.74	1.000
	IDO2	-2.11	0.472	-0.32	1.000
	PTGS2	-2.33	0.357	-0.81	1.000

[a] Benjamini-Yekuteli adjusted p value

[b] D1 = day 1

[c] D3 = day 3

Supplemental Table S5: Pathway enrichment analysis

Analysis	Pathway[a]	FDR p value
**D1 genes associated with one or more antibody responses**		
**g:profiler[b] GO:BP[c]**	cytokine-mediated signaling pathway	1.95E-07
	defense response	1.63E-06
	cellular response to cytokine stimulus	3.18E-06
	inflammatory response	3.24E-06
	cell surface receptor signaling pathway	7.04E-06
	response to cytokine	1.06E-06
	regulation of immune system process	1.51E-05
	immune system process	3.80E-05
	signal transduction	9.73E-05
	immune response	0.0001
**Cytoscape[d] GO:BP**	cytokine-mediated signaling pathway	4.93E-12
	defense response	1.43E-11
	immune system process	2.24E-10
	immune response	2.24E-10
	regulation of immune system process	1.35E-09
	cell surface receptor signaling pathway	1.35E-09
	inflammatory response	1.87E-09
	response to organic substance	1.96E-08
	response to chemical	4.51E-08
	response to external stimulus	2.55E-07
**Cytoscape KEGG[e]**	cytokine-cytokine receptor interaction	2.63E-09
	influenza A	6.27E-08
	hematopoietic cell lineage	7.22E-08
	JAK-STAT signaling pathway	1.39E-06
	Viral protein interaction with cytokine and cytokine receptor	4.22E-06
	Rheumatoid arthritis	1.34E-04
	IL-17 signaling pathway	1.50E-04
	TH17 cell differentiation	1.80E-04
	TNF signaling pathway	2.40E-04
	Herpes simplex virus 1 infection	3.00E-04
	(42)[f] Toll-like receptor signaling	0.0413
**D1 genes associated with ADCC responses**		
**Cytoscape GO:BP**	cytokine-mediated signaling pathway	2.71E-05
	inflammatory response	1.70E-04
	defense response	4.70E-04
	cellular response to organic substance	8.00E-04
	immune system process	9.90E-04
	immune response	0.0012
	positive regulation of nmda glutamate receptor activity	0.0061
	regulation of immune system process	0.0147
	positive regulation of immune system process	0.0234
	regulation of cell adhesion	0.0234
**Cytoscape KEGG**	cytokine-cytokine receptor interaction	2.62E-07
	Leishmaniasis	6.90E-04
	Viral protein interaction with cytokine and cytokine receptor	8.10E-04
	hematopoietic cell lineage	8.10E-04
	IL-17 signaling pathway	8.10E-04
	Th1 and Th2 cell differentiation	8.10E-04
	Th17 cell differentiation	8.10E-04
	Systemic lupus erythematosus	8.10E-04
	Rheumatoid arthritis	8.10E-04
	JAK-STAT signaling pathway	0.0015

ADCC-associated genes - Network 1		
**NetworkAnalyst[g] GO:BP**		
	intracellular protein kinase cascade	4.10E-22
	Immune response	4.10E-22
	regulation of defense response	1.17E-20
	cytokine-mediated signaling pathway	1.17E-20
	regulation of immune response	2.42E-20
	defense response	5.36E-19
	innate immune response	6.67E-19
	positive regulation of defense response	3.77E-17
	regulation of cytokine production	1.07E-16
	regulation of immune system process	1.09E-16
**NetworkAnalyst KEGG[c]**		
	JAK-STAT signaling pathway	6.15E-22
	Measles	4.71E-15
	Pathways in cancer	1.34E-14
	Th1 and Th2 cell differentiation	1.34E-14
	Th17 cell differentiation	8.29E-14
	Kaposi's sarcoma-associated herpesvirus infection	1.00E-10
	Hepatitis	2.03E-10
	Epstein-Barr virus infection	2.03E-10
	Hepatitis B	3.03E-10
	HTLV1 infection	4.84E-10
	(28)[d] Toll-like receptor signaling pathway	6.36E-06
	(38) BCR signaling pathway	0.0002
	(46) NK cell-mediated cytotoxicity	0.0028

[a] the top 10 pathways based on FDR p values are listed
[b] g:profiler: web-based software for pathway analysis (biit.cs.ut.ee)
[c] GO:BP Gene Ontology Biological Processes
[d] Cytoscape: cytoscape.org; Cytoscape version 3.9.1
[e] KEGG Kyoto Encyclopedia of Genes and Genome
[f] (46) rank of pathway in enrichment based on FDR p value
[g] NetworkAnalyst: web-based software for network and pathway analysis (networkanalyst.ca)

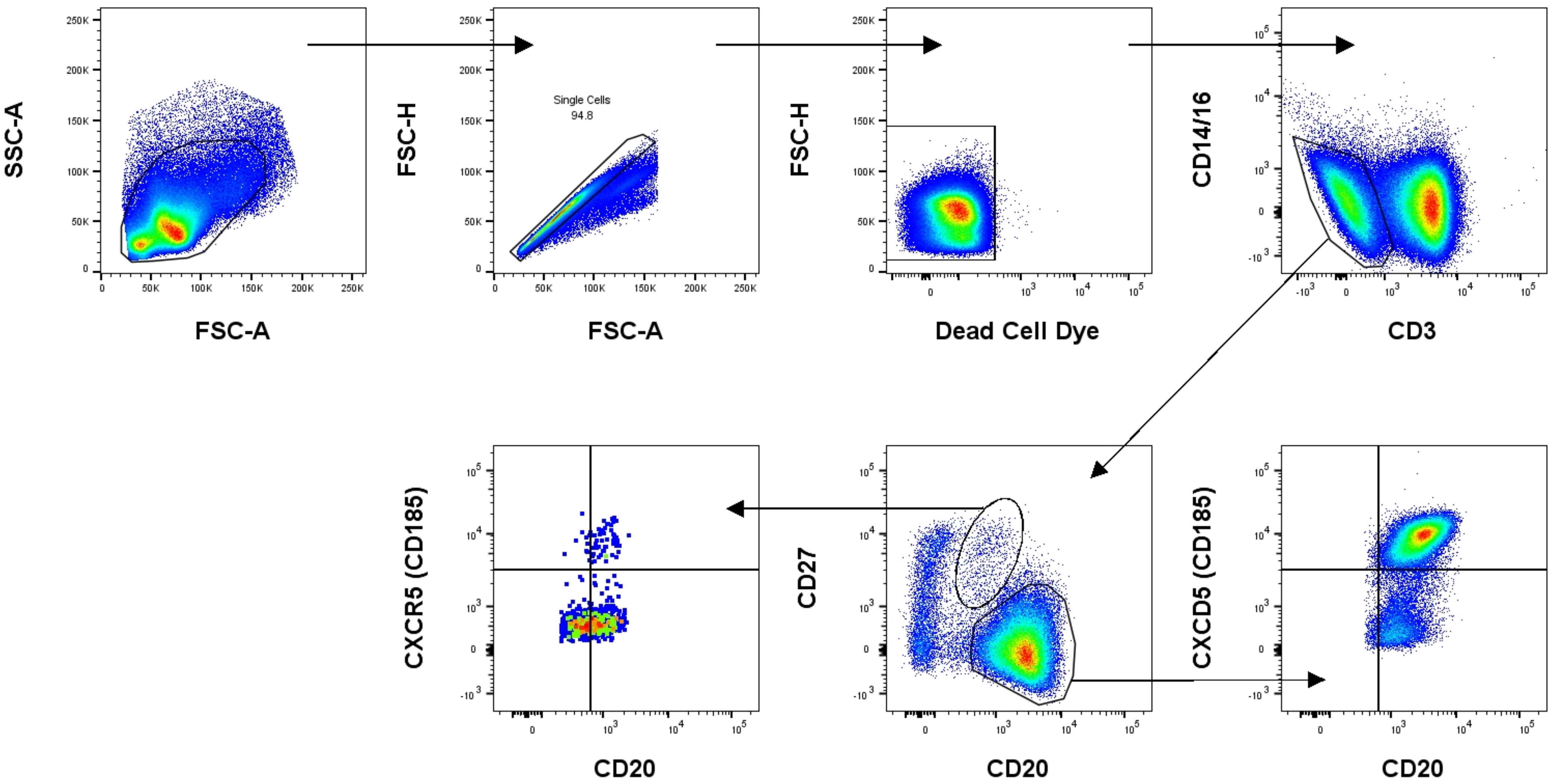
SSC-A
FSC-A
FSC-H
FSC-A
Single Cells
94.8
FSC-H
Dead Cell Dye
CD14/16
CD3
CXCR5 (CD185)
CD20
CD27
CD20
CXCD5 (CD185)
CD20

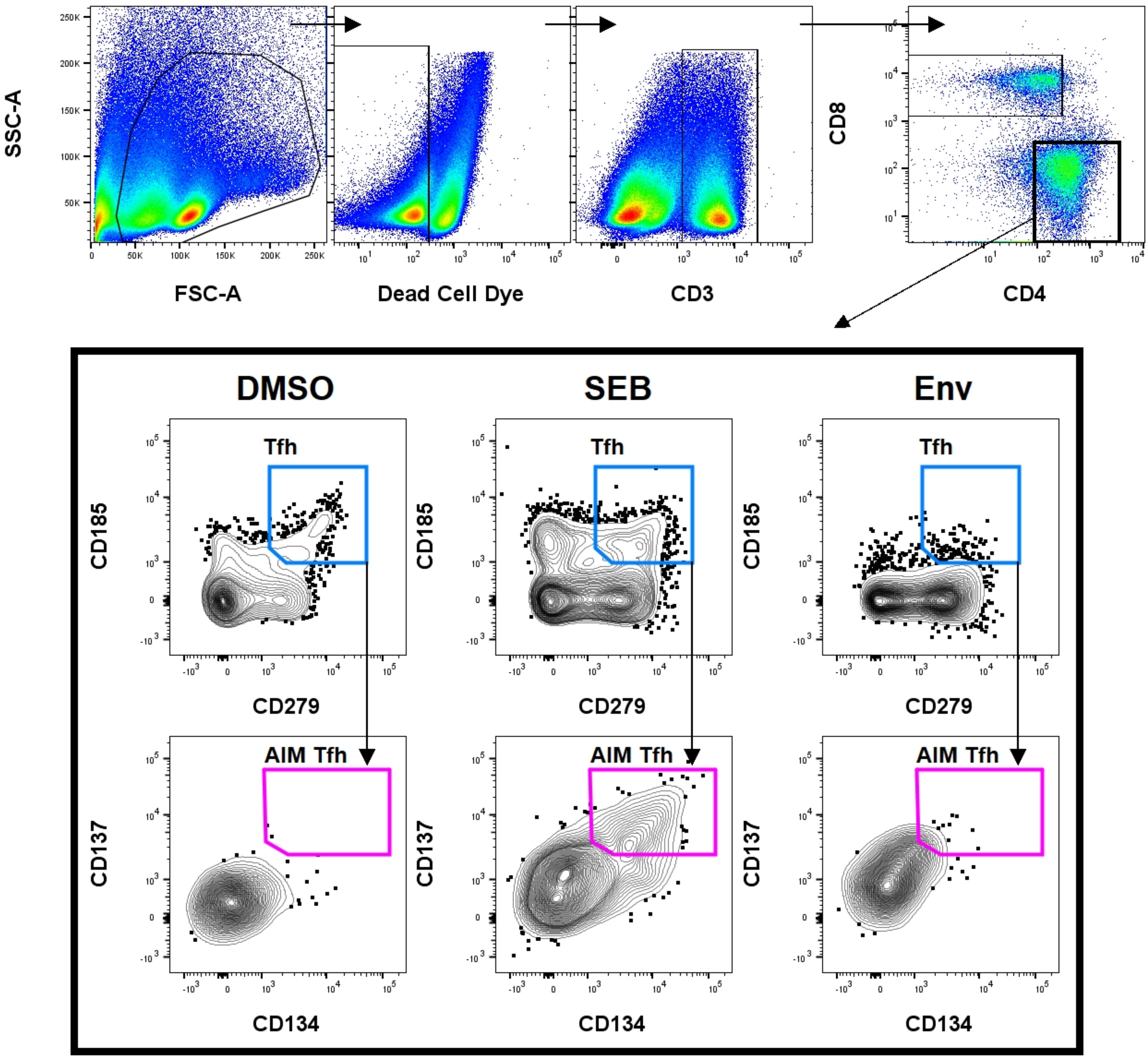

SSC-A
FSC-A
Dead Cell Dye
CD3
CD8
CD4
DMSO
SEB
Env
Tfh
CD185
CD279
AIM Tfh
CD137
CD134

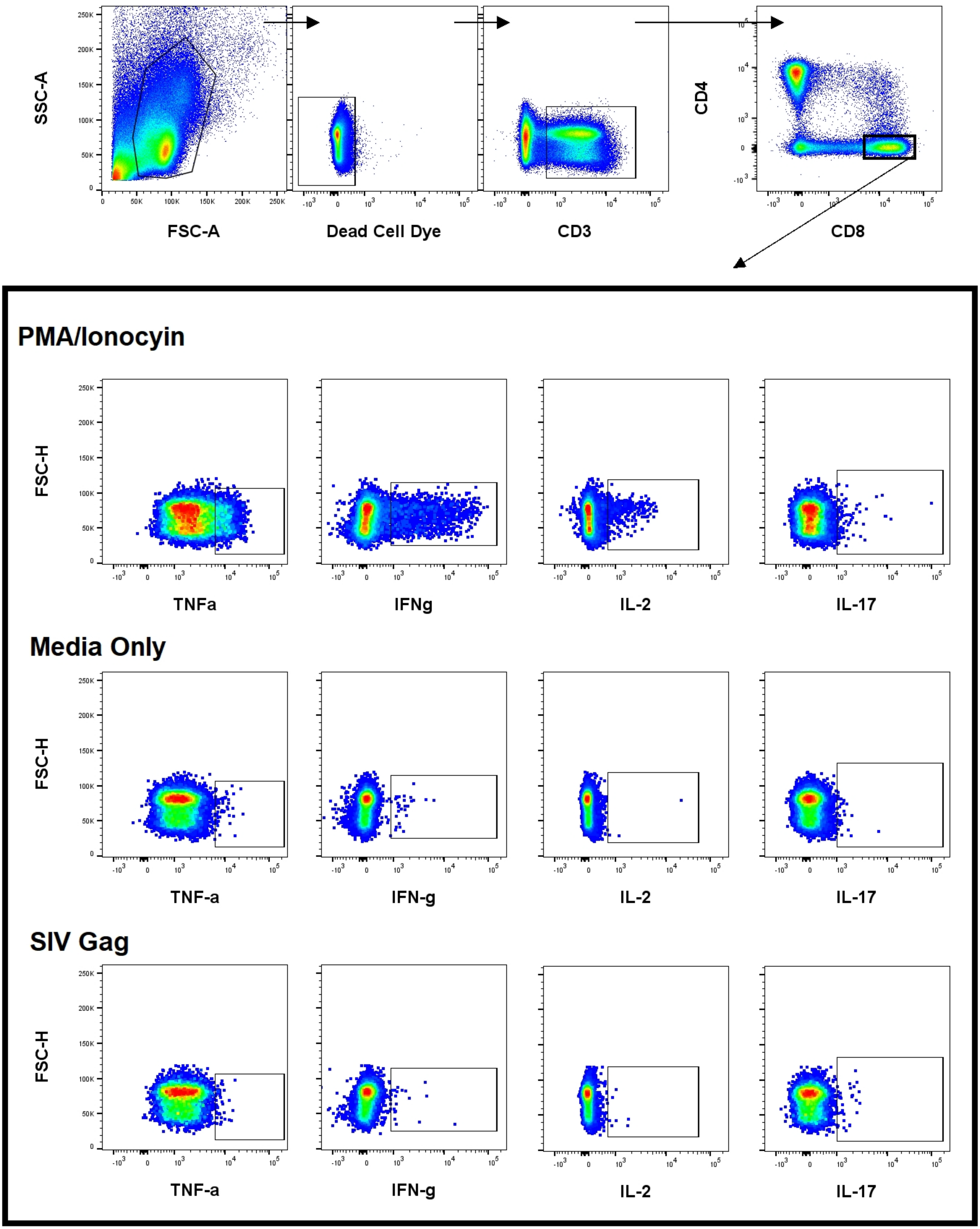

SSC-A
FSC-A
Dead Cell Dye
CD3
CD4
CD8
PMA/Ionocyin
FSC-H
TNFa
IFNg
IL-2
IL-17
Media Only
TNF-a
IFN-g
SIV Gag

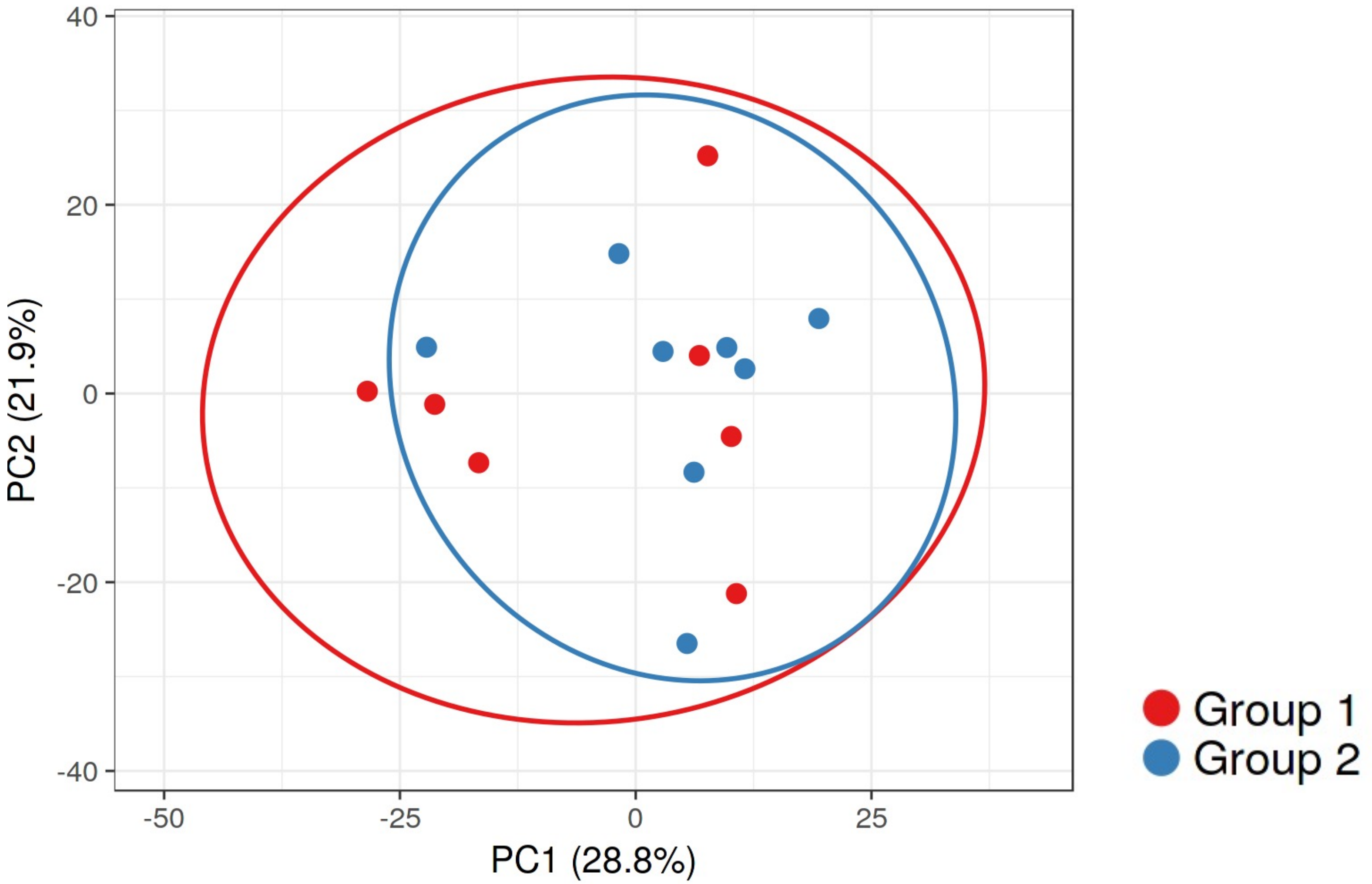

Supplemental Figure S4: Principal component plot of PC1 and PC2 mRNA data of D0 samples from Group 1 (red circles; n=7) and Group 2 (blue circles; n=8). Image was generated using Clustviz.

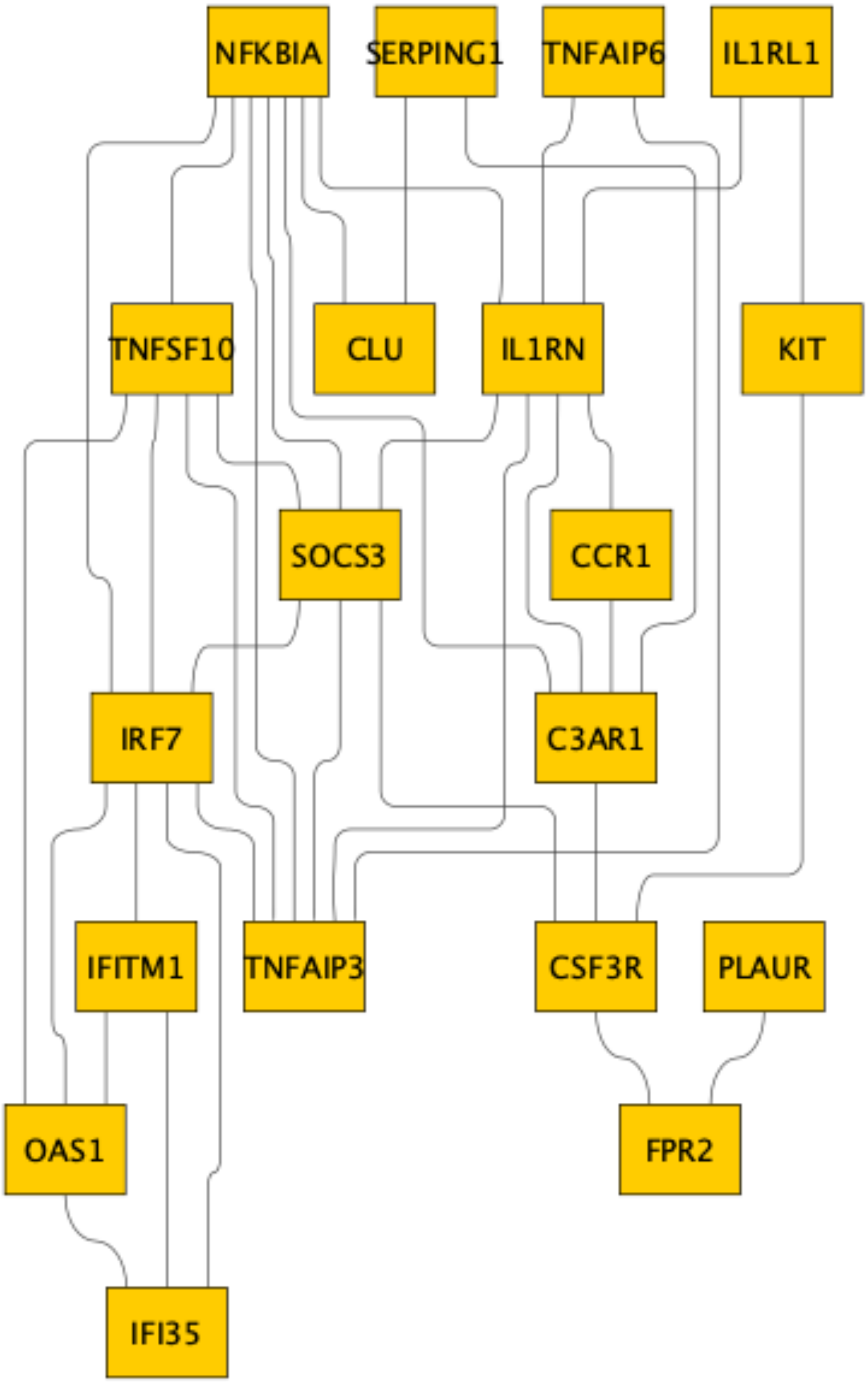
NFKBIA
SERPING1
TNFAIP6
IL1RL1
TNFSF10
CLU
IL1RN
KIT
SOCS3
CCR1
IRF7
C3AR1
IFITM1
TNFAIP3
CSF3R
PLAUR
OAS1
FPR2
IFI35

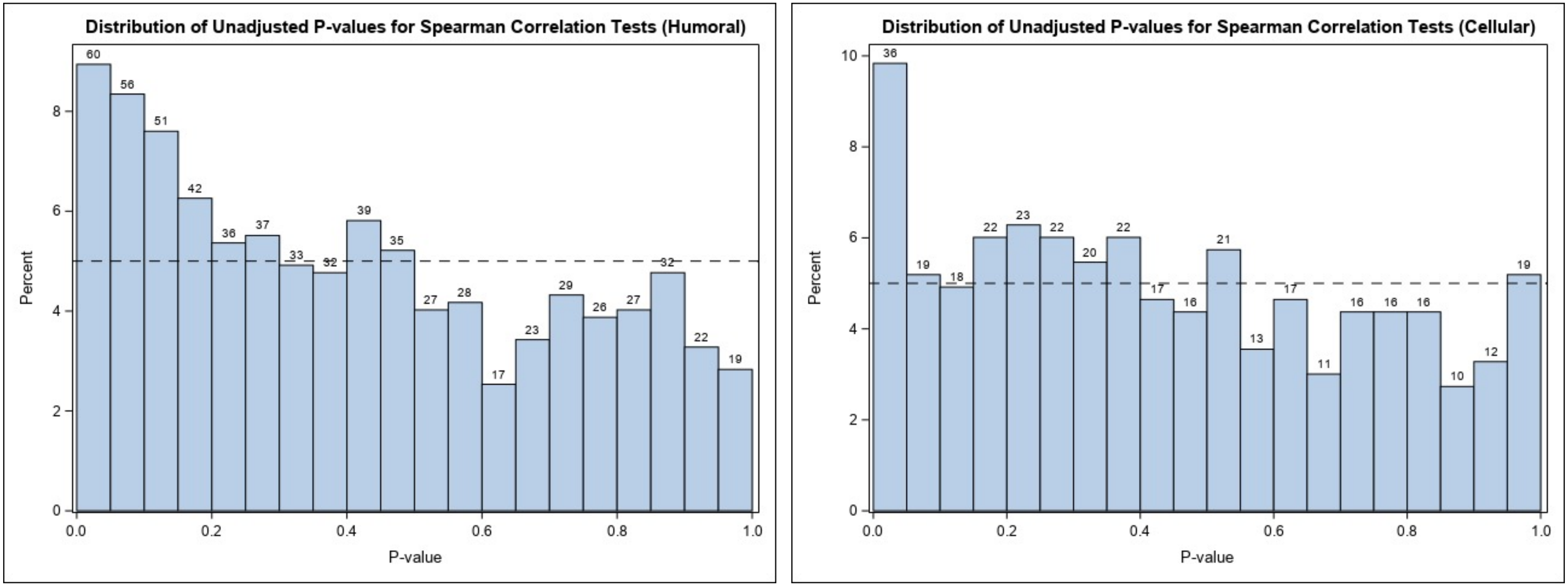

Supplemental Figure S6: Distribution of unadjusted p-values from two-sided tests for Spearman correlations. *Panels A and B* show the distribution of unadjusted p-values from two-sided tests for Spearman correlations (H_a: rho ≠ 0) between differentially regulated transcripts and (A) humoral (Env-specific plasma IgG, ADCC, and neutralizing antibody responses; total tests: n=661) or (B) cellular (memory B and germinal center B cells, T_{FH} cells, and HIV Env- and SIV Gag-specific $CD8^+$ T-cells; total tests: n=366) immune responses. Unadjusted p values in 0.05 increments are listed on the x-axis. The y-axis lists the percentage of unadjusted p values falling into the range of each increment. The number on top of each bar represents the absolute number of tests within each p value range. The dashed line indicates the expected percentage if correlations were randomly distributed.

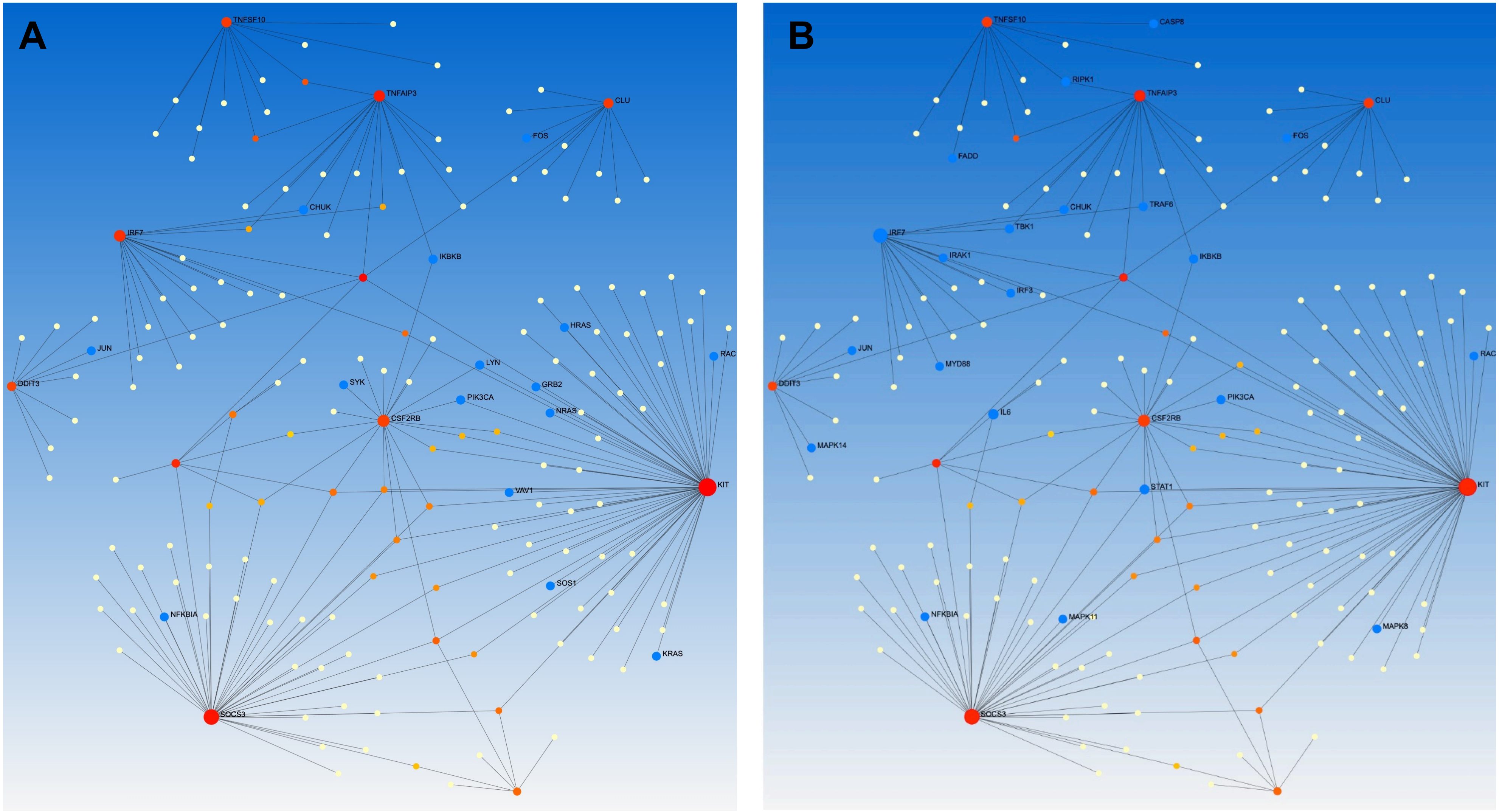

Figure S7: Potential interactions between day 1 induced genes and specific signaling pathways. Genes that were increased on D1 and correlated to vaccine-induced antibody responses were entered into NetworkAnalyst to assemble a network based on the String v11 Human Interactome. Major hubs (nodes) are indicated by red and orange circles. Predicted interactions between the genes are indicated by edges (black lines), with dark blue circles symbolizing predicted interaction partners. In Panels A and B genes that are part of the KEGG B cell receptor or the TLR7/8 signaling pathway, respectively, are represented by light blue circles.
